# Dose-response effects and mechanistic pathways linking physical exercise to brain volume and cognition: a systematic review and meta-analysis of randomized controlled trials

**DOI:** 10.1186/s11556-025-00398-3

**Published:** 2026-01-05

**Authors:** Geng Li, Chengzhen Liu, Antao Chen

**Affiliations:** 1 Brain Health Institute, National Center for Mental Disorders, Shanghai Mental Health Center, Shanghai Jiao Tong University School of Medicine and School of Psychology, Shanghai, China; 2 School of Psychology, Research Center for Exercise and Brain Science, Shanghai University of Sport, Shanghai, China; 3 Faculty of Psychology, Southwest University, Chongqing, China

**Keywords:** Physical exercise, Brain volume, Dose-response effects, Cognitive function

## Abstract

**Background:**

The dose-response effects of physical exercise on brain volume remain insufficiently understood, and it is yet to be established whether exercise-induced relative changes in brain volume translate into improvements in cognitive function. Furthermore, it remains unclear whether specific physical adaptations are associated with both exercise-related changes in brain volume and cognitive outcomes.

**Methods:**

To address these gaps, we conducted a pre-registered systematic review and multilevel meta-analysis of 59 randomized controlled trials comprising a total of 5,099 participants. Comprehensive literature searches were carried out in PubMed, PsycINFO, Embase, and Web of Science up to August 2024. The meta-analysis examined overall effects of physical exercise on brain volume, investigated dose-response effects across relevant moderators, and explored potential mediation pathways linking exercise-induced relative changes in brain volume to cognitive outcomes.

**Results:**

This study found that physical exercise had a significant effect on brain volume. Dose-response analyses further revealed that this effect was more pronounced among individuals of older age, with longer intervention durations and higher compliance. Exercise sessions lasting 40–60 min were associated with the most robust effects on brain volume. Post-hoc analyses suggest that these effects are more likely attributable to the preservation of brain volume rather than substantial volumetric increases. In addition, regression analysis demonstrated a significant positive association between exercise-induced relative changes in brain volume and cognitive function. Moreover, mediation analyses indicated that exercise-induced physical adaptations in muscle function, motor performance, and cardiorespiratory fitness were indirectly associated with cognitive performance through their effects on relative brain volume change.

**Conclusions:**

These findings indicate that physical exercise exerts beneficial effects on brain volume, particularly under conditions such as older age, sufficient exercise duration, and high compliance. Exercise-induced relative changes in brain volume were significantly associated with cognitive function and served as a mediator between physical adaptations and cognitive outcomes. Together, these results provide a mechanistic foundation and practical insights for designing targeted exercise interventions to promote brain health in aging populations.

**Trial registration:**

PROSPERO CRD42024525635.

**Supplementary Information:**

The online version contains supplementary material available at 10.1186/s11556-025-00398-3.

## Background

Brain volume reflects an individual’s ability to adapt to both natural and social environments, exhibiting significant neuroplasticity across the lifespan [1–3]. Although reductions in brain volume during early developmental stages may reflect normal neurodevelopmental processes such as synaptic pruning, age-related declines are typically associated with cognitive impairment [4] and an increased risk of neurodegenerative diseases such as Alzheimer’s disease [5]. These structural changes not only heighten individuals’ dependence on caregivers but also contribute to rising healthcare and long-term care costs, thereby intensifying the economic burden on both individuals and society as a whole [6]. Given these concerns, identifying effective interventions to maintain brain volume is of critical importance.

In response, research on non-pharmacological interventions for preserving brain volume has expanded significantly, with a particular focus on physical exercise. This growing interest stems from the fact that physical exercise is a widely accessible and cost-effective intervention that demands minimal specialized equipment, allowing for broad applicability across diverse populations and settings [7, 8]. Recent studies found that physical exercise significantly reduces the risk of mild cognitive impairment and dementia [9], potentially through its impact on brain volume [10]. Mechanistically, physical exercise promotes neuroplasticity by stimulating neurotrophic factors and facilitating synaptogenesis, axonal growth, dendritic branching, and angiogenesis [11]. However, the relatively small sample sizes in individual studies may contribute to variability in the findings, emphasizing the need for comprehensive synthesis to ensure the robustness of these effects.

Although meta-analyses are essential for synthesizing findings in this area, substantial heterogeneity persists across previous studies [12–14]. For instance, Balbim et al. [15] reported no significant effects of aerobic exercise on brain volume in healthy individuals, whereas Wilckens et al. [16] found significant effects when interventions lasted more than 24 weeks and involved less than 150 min of exercise per week. This heterogeneity may arise from variations in population characteristics and intervention designs [17]. These findings suggest that the effects of physical exercise on brain volume may be selective or follow a dose-response pattern. For example, the Acute Exercise Effect Hypothesis posits that exercise intensity is a critical determinant, likely due to the increased secretion of neurotrophic factors associated with higher-intensity activity [18, 19].

Additionally, the Scaffolding Theory of Aging and Cognition (STAC) suggests that as gray and white matter decline with age, physical exercise promotes compensatory “scaffolding” mechanisms, mitigating brain volume loss and associated cognitive decline [20–22]. This underscores age as a key factor influencing the effects of exercise. Complementing this, the Time-dependent Effect Hypothesis argues that the benefits of physical exercise on brain volume are cumulative, intensifying progressively with longer exercise lengths [23], emphasizing exercise length as another critical determinant. Taken together, these perspectives highlight the urgent need for a robust, evidence-based theoretical framework to address inconsistencies in prior research on exercise-induced relative brain volume changes.

Beyond its theoretical contributions, focusing solely on changes in brain volume without examining their relationship to cognitive outcomes limits the practical significance of such findings [24]. While physical exercise has been shown to promote both brain volume and cognitive function, these effects are often attributed to underlying physiological mechanisms [25–28]. However, little attention has been given to the potential mediating role of specific physical adaptations in linking physical exercise to brain structural changes and, ultimately, cognitive enhancement. Understanding whether and how exercise-induced relative brain volume changes translate into cognitive benefits is essential for advancing mechanistic insight and optimizing real-world interventions.

To address these gaps, the present pre-registered systematic review and multilevel meta-analysis aims to provide quantitative evidence on: (1) the overall effect of physical exercise on relative brain volume change; (2) the dose-response relationship between physical exercise and relative brain volume change across key moderators; (3) the association between exercise-induced relative changes in brain volume and cognitive improvements; and (4) whether improvements in muscle function, motor performance, and cardiorespiratory fitness mediate the effects of physical exercise on brain volume and, subsequently, cognitive function.

## Methods

This systematic review and meta-analysis were conducted based on a predefined protocol [29], which is registered on PROSPERO (CRD42024525635). A PRISMA checklist is available in Supplementary Table S1.

### Search strategy

A systematic search was performed in PubMed, Web of Science, PsycINFO, and Embase through August 2024. Building on previous meta-analyses [12] and incorporating extended work on physical exercise [30], we employed a uniform and transparent search strategy across all databases to enhance reproducibility [31]. The search terms included: (“physical activity” OR “exercise” OR “running” OR “physical training” OR “walking” OR “yoga” OR “baduanjin” OR “tai ji” OR “tai chi”) AND (“neuroimaging” OR “brain volume” OR “brain change” OR “MRI” OR “magnetic resonance imaging”) AND (“random*” OR “RCT”). Some other related terms were explored during the pilot phase but were not included as separate keywords due to the extremely limited number of relevant studies reporting neuroimaging outcomes. Based on the inclusion criteria, two researchers (GL and CL) independently screened titles and abstracts. Full texts of potentially eligible articles were then retrieved and assessed for final eligibility. Discrepancies were resolved through discussion with a third researcher (AC).

### Inclusion and exclusion criteria

This study aims to investigate differences in relative brain volume changes between physical exercise groups and control groups before and after the intervention. The inclusion criteria are as follows: (1) peer-reviewed publications, (2) available in English, (3) RCTs, (4) interventions involving an exclusive physical exercise group, (5) control groups that were either passive (e.g., waitlist or no-contact) or active, involving low-intensity interventions (e.g., stretching, toning, balance training), (6) any outcomes related to brain volume (e.g., global or regional volumes, gray or white matter volume) assessed immediately post-intervention, mid-intervention, or during a follow-up period, and (7) sufficient data to calculate effect sizes (e.g., sample size, mean/median, and standard deviation for pre- and post-intervention, mid-intervention, or follow-up assessments in both groups) or effect measures such as Cohen’ s d, Hedges’ g, or mean difference.

The exclusion criteria are as follows: (1) non-RCTs, (2) animal studies, (3) combined intervention groups (e.g., physical exercise combined with cognitive intervention), and (4) inappropriate control groups (e.g., cognitive training). Additionally, GL and CL directly requested data from authors when it was not provided in the published articles. Studies lacking sufficient data to compute effect sizes were excluded. To minimize bias, we avoided including multiple studies reporting on the same sample or results derived from previously published subsets or databases [32].

### Methodological quality assessment and grading of the evidence

Two independent researchers (GL and CL) assessed the reliability and quality of the included meta-analyses using the Cochrane risk-of-bias tool for randomized trials [33]. This tool classifies the risk of bias as ‘low,’ ‘high,’ or ‘unclear’ across six trial design aspects: sequence generation, allocation concealment, blinding of outcome assessment, incomplete outcome data, selective outcome reporting, and other potential biases.

### Study selection and data extraction

The search, screening, and data extraction were independently performed by two researchers (GL and CL) and verified by a third researcher (AC). Data were extracted using a standardized form, including effect size information (e.g., sample size, mean or median, and standard deviation), or alternative metrics such as Cohen’s d, Hedges’ g, or mean differences, for outcomes related to brain volume, cognitive function, and exercise-induced physical adaptations. Additional variables included participant characteristics (e.g., average age, sex ratio, and clinical status), exercise parameters (e.g., duration, frequency, length, compliance, intensity, and compliance), and study assessment time point. Detailed operational definitions of primary variables and moderators are provided in Supplementary Table S2 and S3.

### Data synthesis and statistical approach

Supplementary Information provides a detailed description of our data synthesis and statistical methodology. Briefly, we conducted a multilevel meta-analysis to assess the overall effects of physical exercise on relative brain volume change and to identify moderators of intervention efficacy. A random-effects model using Restricted Maximum Likelihood (REML) estimation was employed, with effect sizes expressed as Hedges’ g, where a positive value reflects a relative increase in brain volume in the exercise group compared to controls [34]. Post-hoc analyses pooled the mean changes in brain volume reported by exercise and control groups to determine whether the effects of physical exercise on brain volume were due to an actual increase in brain volume rather than an attenuation of brain volume loss [12]. Dose-response analyses were conducted to assess whether the effects of physical exercise on relative brain volume change were modulated by exercise parameters and participant characteristics. Bayesian mediation analyses were employed to investigate whether exercise-induced physical adaptations mediate the effects of physical exercise on brain volume and, subsequently, cognitive function. Additionally, regression analyses were performed to examine the relationship between relative changes in brain volume and cognitive function. Heterogeneity was evaluated using the I² statistic and the Cochrane Q test, while publication bias was assessed through funnel plots.

## Results

### Study selection

**Fig. 1:**
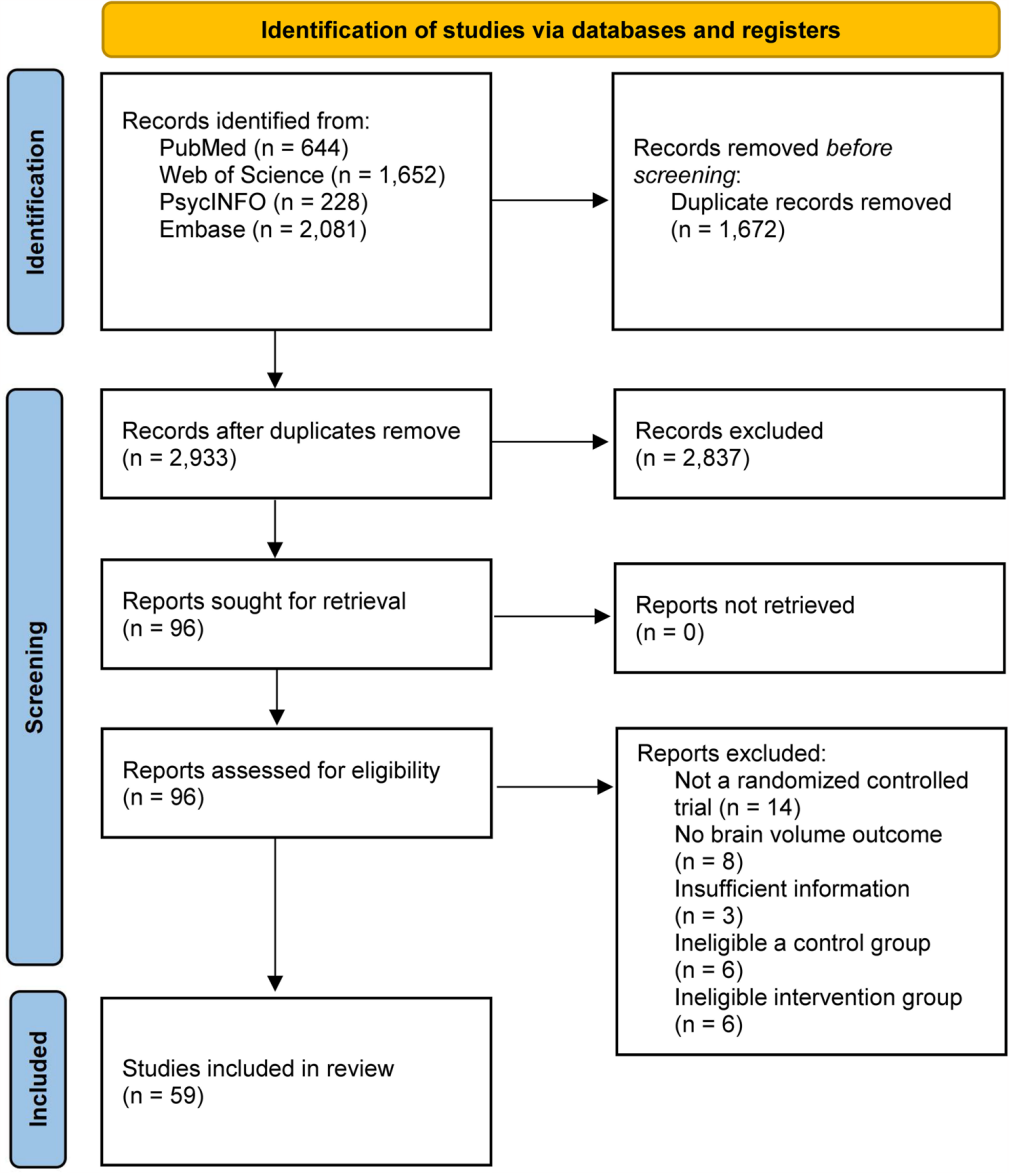
The process study selection shown on PRISMA flow chart

A total of 4,605 studies were initially retrieved from the database searches, with 1,672 duplicates removed. After screening the remaining 2,933 articles by titles and abstracts, 96 articles underwent full-text evaluation. Of these, 59 articles fully met the inclusion criteria for quantitative synthesis. The reference list of included studies is provided in Supplementary Table S4, and reasons for study exclusion are in Supplementary Table S5. Figure 1 outlines the study selection process. A total of 59 RCTs involving 5,099 participants were analyzed to evaluate the effects of physical exercise on relative brain volume change. On average, interventions lasted 30 weeks, with a follow-up period of 36 weeks, comprising sessions of 54.34 min each, conducted 2.57 times per week, as detailed in Supplementary Table S6.

### Risk of bias in included studies

**Fig. 2:**
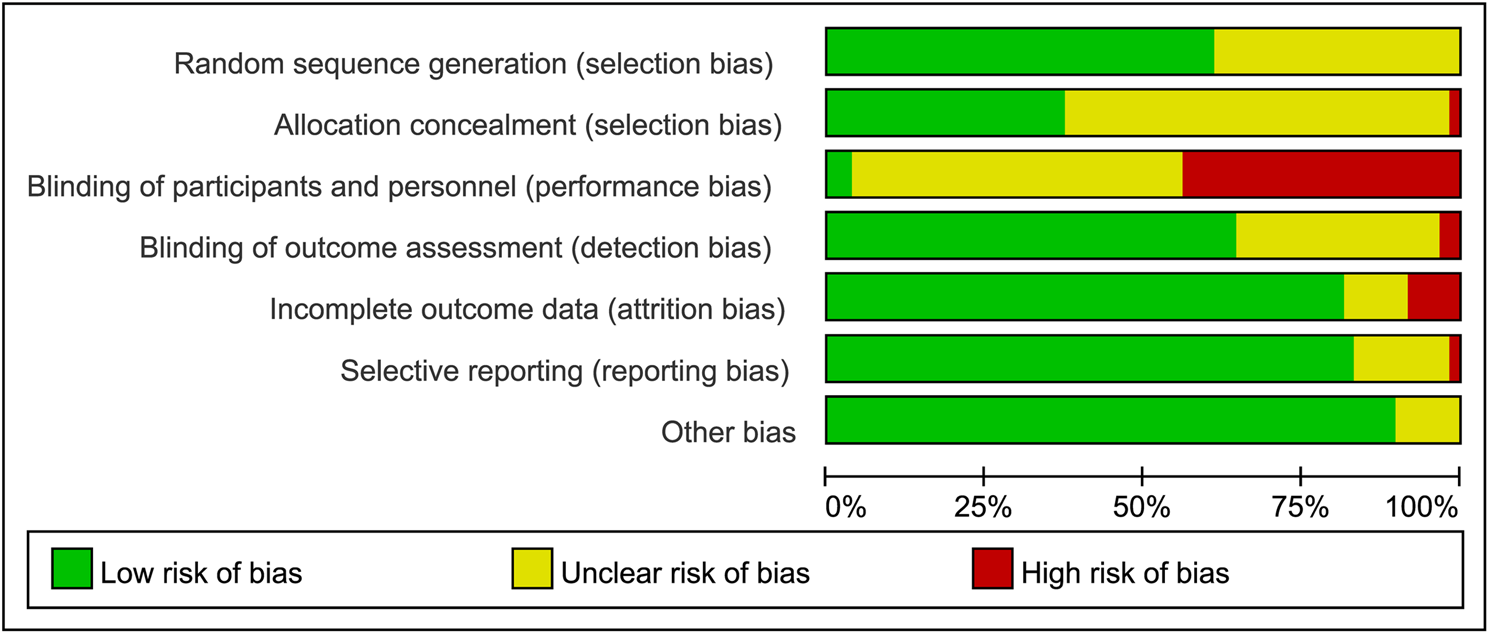
Risk of bias ratio diagram

Although all included studies were RCTs, the gold standard for intervention research, performance bias from inadequate participant blinding emerged as the most significant risk (Fig. 2). Only two studies were rated as low risk because they reported participant blinding, while the remaining studies were assessed as having unclear or high risk due to insufficient details on blinding procedures. In addition, studies generally exhibited a low risk of other bias (89%), selective outcome reporting (83%), and incomplete outcome data (81%), but lower ratings were found for blinding of outcome assessment (77%), random sequence generation (61%), and allocation concealment (44%). Detailed risk of bias assessments for each study are provided in Supplementary Table S7.

### Meta-analysis

The meta-analysis revealed that physical exercise had a significant effect on relative brain volume changes compared to the control group (Hedges’ g = 0.10, 95% CI [0.05, 0.15], *p* < 0.0001, I² = 22.83%).

#### Dose-response analysis

**Fig. 3:**
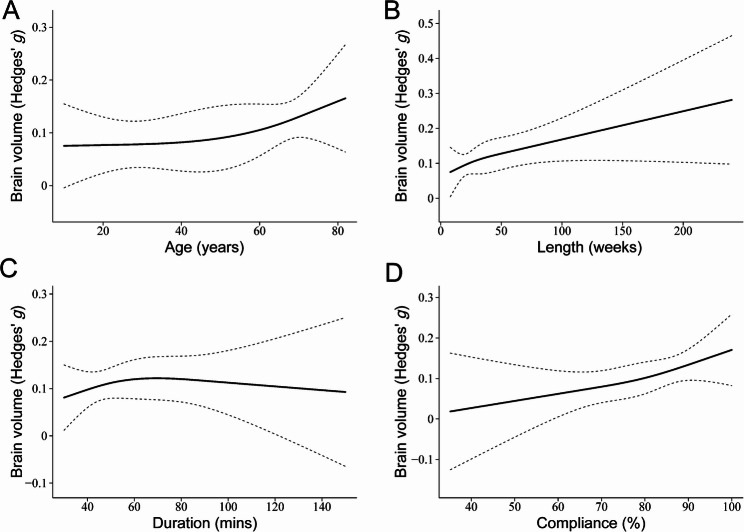
Dose-response analysis exploring the effect of physical exercise on brain volume by (**a**) participant age, (**b**) exercise length, (**c**) duration, and (**d**) compliance

The dose-response analysis indicated that greater participant age, longer overall exercise duration, and higher compliance rates were associated with stronger effects of physical exercise on brain volume (Fig. 3a, b, and d). Notably, the analysis revealed that exercise sessions lasting approximately 40 to 60 min were associated with the most pronounced effects (Fig. 3c). In contrast, no significant effects were observed for participant sex distribution, exercise frequency, or exercise intensity.

#### Post-hoc analyses

Post-hoc analyses revealed no significant increase in brain volume among exercise groups (Hedges’ g = -0.02, 95% CI [-0.10, 0.06], *p* > 0.05), but a significant decrease in brain volume among control groups (Hedges’ g = -0.12, 95% CI [-0.21, -0.02], *p* < 0.05). These findings suggest that the effects observed in this study are more likely attributable to the maintenance of brain volume rather than a substantial increase.

#### Regression analysis

**Fig. 4:**
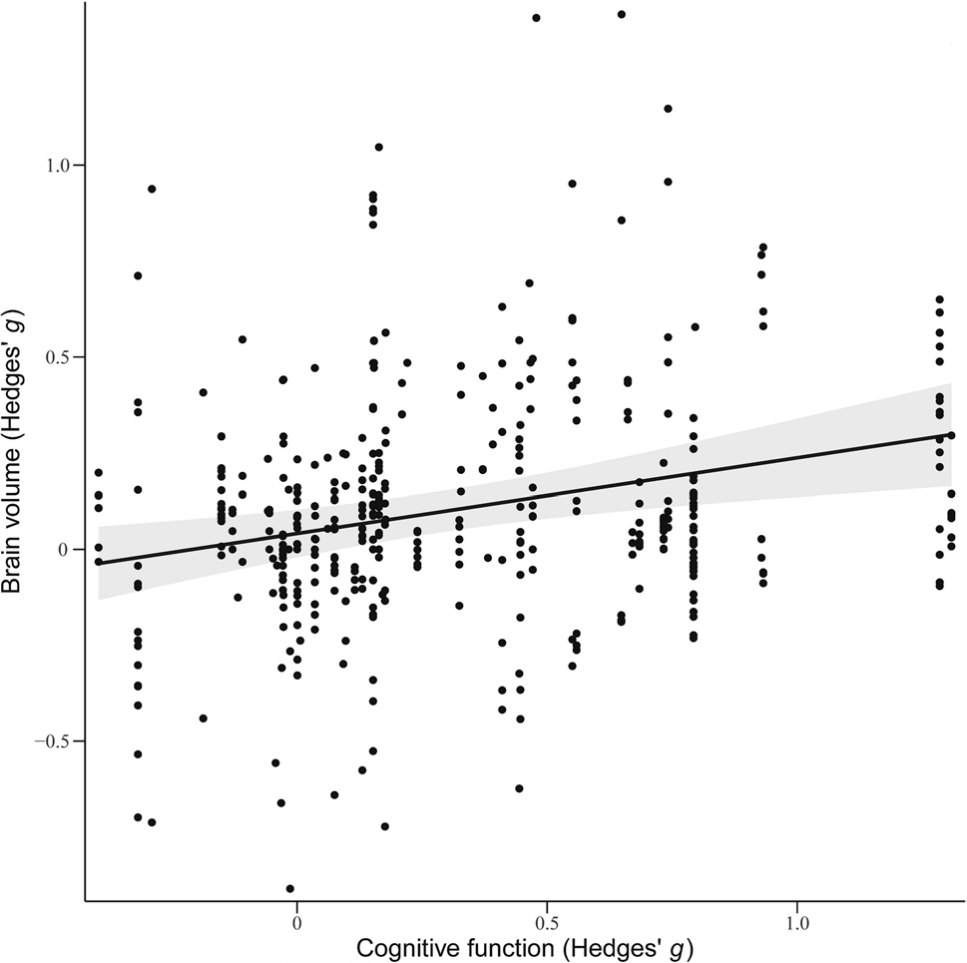
Regression analysis linking exercise-induced relative changes in brain volume to cognitive function. Note: Cognitive function refers to global cognition as operationalized in this study. Detailed definitions and the specific measures included are provided in Supplementary Table S2. A total of 44 studies reporting cognitive outcomes were included in the analysis

Regression analysis demonstrated a significant positive association between physical exercise-induced relative changes in brain volume and improvements in cognitive function (β = 0.20, SE = 0.06, *p* < 0.01; see Fig. 4).

#### Bayesian mediation analyses

**Fig. 5:**
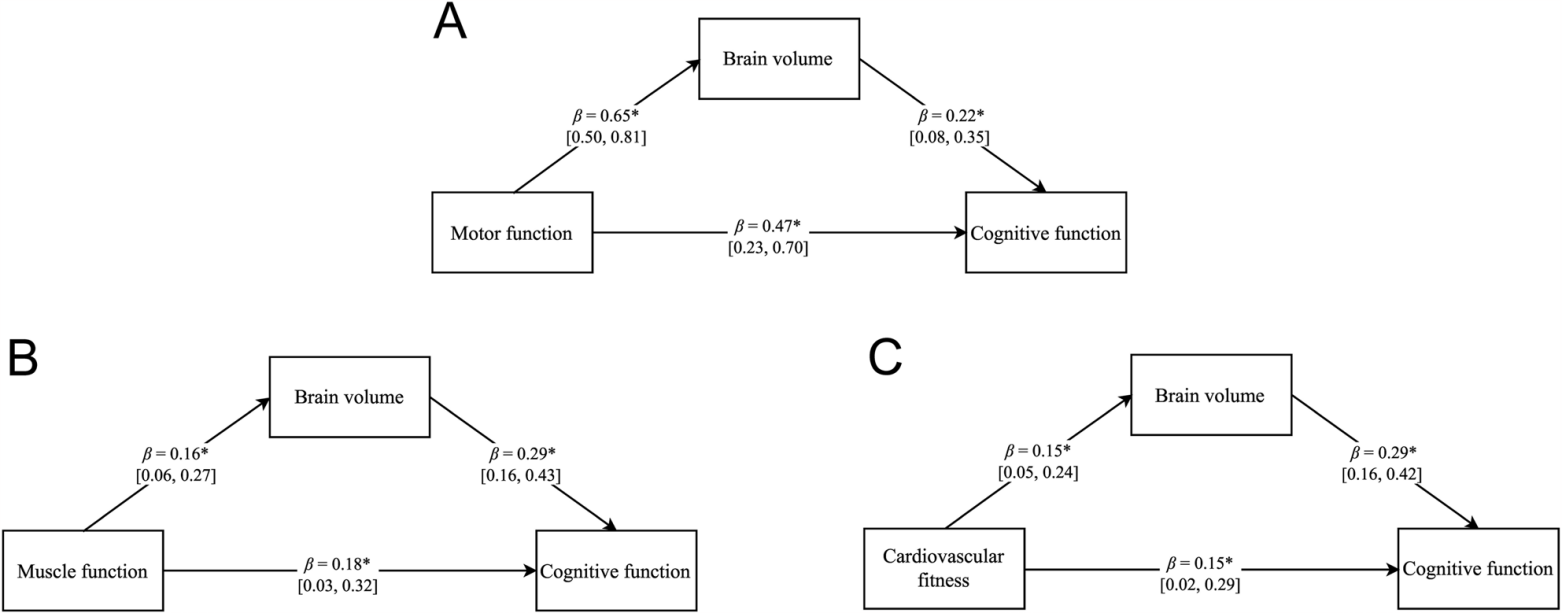
Bayesian mediation analyses assessing whether the associations between (**a**) motor performance, (**b**) muscle function, and (**c**) cardiorespiratory fitness and cognitive function are mediated by brain volume. Note: Detailed definitions and the specific measures for each domain are provided in Supplementary Table S2

Bayesian mediation analyses showed that exercise-induced relative changes in brain volume partially mediated the links between physical adaptations and cognitive function. Specifically, mediation effects were observed for motor performance, muscle function, and cardiorespiratory fitness (Fig. 5).

### Publication bias

Publication bias was assessed using a funnel plot (Supplementary Figure S1). The plot showed a symmetrical distribution of effect sizes around the mean, with no apparent skew or asymmetry, suggesting no substantial publication bias across the included studies.

### Sensitivity analyses

Sensitivity analyses were conducted by including brain volume measurement type as a covariate in the meta-analytic model. The overall effect of physical exercise on brain volume remained significant (Hedges’ g = 0.10, 95% CI [0.05, 0.15], *p* < 0.0001), indicating that the main findings were robust across different types of brain volume metrics. Additional dose-response analyses stratified by clinical versus non-clinical populations revealed a significant difference in the association between age and exercise-induced relative changes in brain volume (Supplementary Figure S2). In contrast, the other dose-response findings remained stable, indicating that these results were not substantially influenced by the clinical status of the sample.

## Discussion

The primary objectives of this study were to address existing gaps by providing quantitative evidence on the overall effect of physical exercise on brain volume, characterizing the dose-response effects, examining whether exercise-induced changes in brain volume are associated with improvements in cognitive function, and analyzing the underlying mechanisms that may account for these effects. Overall, physical exercise had a significant positive effect on brain volume. Dose-response analyses further revealed that greater participant age, longer overall intervention duration, and higher compliance rates were associated with stronger effects in relative brain volume changes. Exercise sessions lasting approximately 40 to 60 min produced the most pronounced outcomes. Furthermore, changes in brain volume resulting from physical exercise were associated with improvements in cognitive function. Finally, brain volume was found to partially mediate the relationships between cognitive function and physical adaptations, including motor performance, muscle function, and cardiorespiratory fitness.

These findings underscore the substantial heterogeneity reported in previous meta-analyses [12–14] and highlight a critical gap in the literature: most prior research has focused on whether physical exercise affects relative brain volume changes, without examining the presence of a dose-response effects. In the current study, greater participant age, longer intervention duration, and higher compliance rates were associated with stronger effects, with sessions lasting approximately 40 to 60 min yielding the most pronounced outcomes. This pattern may contrast with the Acute Exercise Effect Hypothesis proposed in earlier work [18, 19]. Post-hoc analyses further suggested that these effects are more likely attributable to the preservation of brain volume rather than a direct increase, lending support to both the STAC and the Time-Dependent Effect Hypothesis. Unlike the meta-analysis by Gogniat et al. [14], which focused exclusively on individuals over 60 years of age with mild cognitive impairment (MCI), the present study imposed no age or clinical status restrictions on the included population. This broader inclusion strategy enabled the identification of dose-response relationships across key moderators, thereby offering more generalizable and detailed insights into how exercise parameters influence relative brain volume changes.

Sensitivity analyses stratified by clinical and non-clinical populations revealed an inverted U-shaped relationship between age and exercise-induced relative changes in brain volume. This pattern may be explained by the relative stability of brain volume in younger adults, who have not yet experienced noticeable atrophy, whereas both children and older adults exhibit greater neuroplastic potential for structural brain changes [35, 36]. The underlying temporal mechanisms likely involve multiple levels of neuroadaptation, ranging from rapid neuronal adjustments (e.g., synaptic remodeling and spine plasticity) to slower processes such as neurogenesis, gliogenesis, and angiogenesis [23, 37]. Gliogenesis involves the generation of glial cells (e.g., astrocytes and oligodendrocytes), which support neuroprotection, myelination, and metabolic function [38]. Physical exercise promotes gliogenesis, enhancing neural repair and resilience [39]. It also stimulates angiogenesis, improving cerebral blood flow and nutrient delivery, thereby facilitating neuroplasticity and preserving brain volume [40]. Additionally, physical exercise may delay β-amyloid deposition, a hallmark of aging, thereby contributing to the preservation of brain volume [41]. Building on the frameworks of the STAC and the Time-Dependent Effect Hypothesis, we propose the Progressive Scaffolding Accumulation Hypothesis to explain our findings and address inconsistencies in previous research on exercise-induced brain volume changes. This hypothesis suggests that physical exercise incrementally mitigates the effects of aging by fostering compensatory brain changes and leveraging cumulative benefits over time.

A key question in this field is whether changes in brain volume are associated with variations in cognitive function, and what factors may account for this relationship. Our study found that relative brain volume changes induced by physical exercise were significantly associated with cognitive function improvements, providing neurobiological evidence to support existing theoretical frameworks [8]. Furthermore, exercise-induced physical adaptations in motor performance, muscle function, and cardiorespiratory fitness were each significantly associated with cognitive improvements through their relationship with brain volume. Epidemiological studies indicate that higher physical activity levels are linked to improved cognitive function in aging [42] and a lower risk of dementia [43]. These associations are partly mediated by structural brain changes, such as slower gray matter atrophy [11], maintained white matter integrity [44], and enhanced synaptic connectivity [45]. However, despite previous research suggesting associations between physical exercise, brain structure, and cognitive function [12–14], the present study is the first to systematically link exercise-induced relative changes in brain volume with cognitive improvements, while incorporating physical adaptations into a mediation framework. This approach provides robust empirical support for the neurobiological pathway connecting physical exercise to cognitive enhancement.

Our findings address inconsistencies in previous research on exercise-induced brain volume changes and offer several promising directions for enhancing future physical exercise interventions. However, these findings must also be interpreted in the context of their limitations. Firstly, implementing blinding in physical exercise interventions is considerably more challenging compared to pharmaceutical trials [46], which may introduce various experimental biases and affect the reliability of the results. Secondly, due to the limited descriptions of intervention protocols in the included studies, our coding of exercise intensity was primarily based on the intended intensity rather than the actual measured intensity, which may have resulted in effect sizes not accurately reflecting the true benefits of different exercise intensities. Moreover, the brain volume relative changes observed in this study may reflect selective structural preservation rather than widespread or nonspecific alterations, potentially influenced by regional vulnerability and tissue-specific plasticity. Given the limited number of available studies, we were unable to conduct systematic analyses across different brain regions and tissue types. Future research should aim to differentiate volumetric changes by region and tissue to better elucidate the neuroprotective mechanisms of physical exercise. Additionally, although our search strategy encompassed several specific forms of physical exercise, some terms were excluded due to the extremely limited number of relevant studies identified during preliminary searches. While we attempted to mitigate this issue by including broad terms, a potential bias in the representativeness of intervention types may still remain. Finally, the limited data prevented us from examining the relationship between improvements in specific cognitive functions and relative changes in distinct brain regions, restricting the specificity of our conclusions.

## Conclusions

This study provides robust evidence that physical exercise exerts selective effects on brain volume, which follow dose-response effects and are significantly associated with cognitive improvements. These effects were more pronounced in older individuals, with longer intervention durations and higher compliance, highlighting the cumulative and age-sensitive benefits of exercise. Moreover, exercise-induced physical adaptations were significantly linked to brain volume and, in turn, cognitive function, suggesting that these adaptations may serve as key mediating pathways. Together, these findings offer mechanistic and theoretical insights into how physical exercise promotes brain health and support the development of optimized intervention strategies aimed at enhancing brain volume and cognitive function across the lifespan.

## Supplementary Information

Supplementary Material 1.

## Data Availability

No datasets were generated or analysed during the current study.
