# Supplementary material for: Dose-response effects and mechanistic pathways linking physical exercise to brain volume and cognition: a systematic review and meta-analysis of randomized controlled trials
Source: Eur Rev Aging Phys Act. 2026 Jan 5;23:6. doi: 10.1186/s11556-025-00398-3 (PMC12870280; doi:10.1186/s11556-025-00398-3)
Supplement: Supplementary file 1 — Supplementary Material 1. [file 11556_2025_398_MOESM1_ESM.docx]

Supplementary Material Contents

[Supplementary Information 1](#_Toc23958)

[Table S1. PRISMA checklist 4](#_Toc23958)

[Table S2. Operational definitions of primary variables 7](#_Toc23958)

[Table S3. Descriptions of moderators 8](#_Toc23958)

[Table S4. Reference list of included studies. 9](#_Toc3750)

[Table S5. Reasons for study exclusion. 1](#_Toc2421)4

[Table S6. Information of included studies 1](#_Toc15234)9

[Table S7. Risk of bias summary](#_Toc15234) 26

[Figure S1. Funnel plot 3](#_Toc921)0

[Figure S2. Sensitivity analysis of dose-response relationships stratified by clinical versus non-clinical cohorts 3](#_Toc921)1

## Supplementary Information

*Data synthesis and statistical approach*

Brain volume serves as a key macrostructural indicator of both neuroplasticity and neurodegeneration [1]. Different types of volumetric changes capture the brain’s multidimensional structural responses to external interventions like physical exercise[2-3]. Several prior systematic reviews and meta-analyses have adopted similar integrative strategies, combining whole brain, tissue-specific (e.g., gray and white matter), and region-specific volumes (e.g., hippocampus, frontal cortex), in order to enhance statistical power and external validity [4]. However, in cases where multiple reports originated from the same participant sample and assessed the same brain volume outcomes, we retained only one study to avoid duplication. If different reports from the same randomized controlled trial (RCT) involved distinct subsamples or focused on different brain regions, they were treated as independent samples and included separately in the analysis.

Given that many original studies report only specific regions or tissue types (e.g., hippocampal gray matter or frontal white matter), restricting inclusion to a single metric such as total brain volume would substantially reduce the available data and limit the generalizability of findings. Therefore, integrating multiple yet related brain volume indicators enables a more comprehensive representation of exercise-related structural brain changes and increases the robustness and interpretability of the results. However, this integrative approach introduces a nested data structure. Many primary studies report multiple effect sizes across different brain regions or volume types, which leads to statistical dependencies that violate the independence assumptions of traditional meta-analytic models and may bias effect estimates [5]. To address this issue, we employed a multilevel meta-analytic model that explicitly accounts for data nesting, improving the precision of effect estimates and reducing systematic bias [6].

In this model, effect sizes (level 1) are nested within samples (level 2), which are further nested within studies (level 3). Analyses were conducted using restricted maximum likelihood (REML) estimation in RStudio (version 1.4.1106) with the metafor package [7-8]. This method provides a statistically robust approach to control for heterogeneity introduced by different brain regions (e.g., whole brain vs. regional) and tissue types (e.g., gray vs. white matter), thereby ensuring the consistency and validity of both statistical and biological interpretations.

Building on prior research [4, 9], we separately aggregated data across different types of brain volume measures and subdomains of cognitive functions. For each domain—brain volume and cognitive function—we calculated an individual standardized effect size, generating composite effect sizes and variances to support subsequent meta-analysis. The intervention effects were quantified using standardized mean differences (Hedges' g), which allowed us to account for differences in measurement units across studies—such as whether brain volume was reported in raw units, normalized to intracranial volume (ICV), or based on region-specific metrics [10].

This method enables the comparison of effect sizes across imaging studies that do not report raw volume differences, while correcting for bias in standardized mean differences and accounting for the impact of small sample sizes [11]. Effect sizes were interpreted as small (≤0.2), medium (0.2-0.8), or large (≥0.8). A positive effect size indicates a relative increase in brain volume in the physical exercise group compared to the control group.

We conducted a dose-response analysis using the rms package, following prior methods [12]. For the Bayesian mediation analyses, we used the brms package in R, which provides a flexible framework for estimating hierarchical Bayesian models via Stan. Each model was run with 4 Markov chains, 2,000 iterations per chain (including 1,000 warm-up iterations), and default weakly informative priors. Convergence was assessed by inspecting trace plots and ensuring that all R̂ (R-hat) values were below 1.01, indicating adequate mixing. Posterior distributions were summarized using mean estimates and 95% credible intervals (CrI). Among all included studies, motor performance, muscle function, and cardiorespiratory fitness were the only exercise-induced physical adaptations with sufficient effect size data (n > 50) to support Bayesian mediation analyses.

To determine whether these effects on brain volume were the result of an actual volume increase, rather than merely sustaining brain volume, post-hoc analyses were conducted to pool the mean changes in brain volume reported in the physical exercise and control groups, following the approach established in previous research [13]. Finally, heterogeneity was assessed using the I^2^ statistic and the Cochrane Q test, with an I^2^ value greater than 50% or a Q test p-value below 0.1 indicating significant heterogeneity among studies [7]. Publication bias was assessed through a funnel plot, which was visually inspected for asymmetry or deviations from the expected distribution as indicators of potential bias [14]. Sensitivity analyses were conducted from two perspectives to test the robustness of the main findings. First, brain volume measurement type (e.g., whole brain, regional volumes, gray matter, or white matter) was included as a covariate in the meta-analytic model to determine whether the overall effect of physical exercise on brain volume remained statistically significant after controlling for variability in measurement. Second, stratified dose-response analyses were performed by dividing the sample into clinical and non-clinical populations. This allowed for the examination of whether the dose-response patterns differed significantly between these two groups.

**Table S1. PRISMA checklist.**

| **Section/topic** | **#** | **Checklist item** | **Reported on**  **page #** |
| --- | --- | --- | --- |
| **TITLE** |  |  |  |
| Title | 1 | Identify the report as a systematic review, meta-analysis, or both. | 1 |
| **ABSTRACT** |  |  |  |
| Structured summary | 2 | Provide a structured summary including, as applicable: background; objectives; data sources; study eligibility criteria, participants, and interventions; study appraisal and synthesis methods; results; limitations; conclusions and implications of key findings; systematic review registration number. | 1 |
| **INTRODUCTION** |  |  |  |
| Rationale | 3 | Describe the rationale for the review in the context of what is already known. | 2-3 |
| Objectives | 4 | Provide an explicit statement of questions being addressed with reference to participants, interventions, comparisons, outcomes, and study design (PICOS). | 3 |
| **METHODS** |  |  |  |
| Protocol and  registration | 5 | Indicate if a review protocol exists, if and where it can be accessed (e.g., Web address), and, if available, provide registration information including registration number. | 3 |
| Eligibility criteria | 6 | Specify study characteristics (e.g., PICOS, length of follow-up) and report characteristics (e.g., years considered, language, publication status) used as criteria for eligibility, giving rationale. | 4 |
| Information sources | 7 | Describe all information sources (e.g., databases with dates of coverage, contact with study authors to identify additional studies) in the search and date last searched. | 4-5 |
| Search | 8 | Present full electronic search strategy for at least one database, including any limits used, such that it could be repeated. | 4-5 |
| Study selection | 9 | State the process for selecting studies (i.e., screening, eligibility, included in systematic review, and, if applicable, included in the meta-analysis). | 5 |
| Data collection  process | 10 | Describe method of data extraction from reports (e.g., piloted forms, independently, in duplicate) and any processes for obtaining and confirming data from investigators. | 5-6 |
| Data items | 11 | List and define all variables for which data were sought (e.g., PICOS, funding sources) and any  assumptions and simplifications made. | Supplementary material Table S1 |
| Risk of bias in  individual studies | 12 | Describe methods used for assessing risk of bias of individual studies (including specification of whether this was done at the study or outcome level), and how this information is to be used in any data synthesis. | 5-6 |
| Summary measures | 13 | State the principal summary measures (e.g., risk ratio, difference in means). | Supplementary Information |
| Synthesis of results | 14 | Describe the methods of handling data and combining results of studies, if done, including measures of consistency (e.g., I^2^) for each meta-analysis. | Supplementary Information |
| Risk of bias across  studies | 15 | Specify any assessment of risk of bias that may affect the cumulative evidence (e.g., publication bias, selective reporting within studies). | 5-6 |
| Additional analyses | 16 | Describe methods of additional analyses (e.g., sensitivity or subgroup analyses, meta-regression), if done, indicating which were pre-specified. | Supplementary Information |
| **RESULTS** |  |  |  |
| Study selection | 17 | Give numbers of studies screened, assessed for eligibility, and included in the review, with reasons for exclusions at each stage, ideally with a flow diagram. | 6 |
| Study characteristics | 18 | For each study, present characteristics for which data were extracted (e.g., study size, PICOS, follow-up period) and provide the citations. | Supplementary material Table S6 |
| Risk of bias within  studies | 19 | Present data on risk of bias of each study and, if available, any outcome level assessment (see item 12). | 7 |
| Results of individual  studies | 20 | For all outcomes considered (benefits or harms), present, for each study: (a) simple summary data for each intervention group (b) effect estimates and confidence intervals, ideally with a forest plot. | 8-9 |
| Synthesis of results | 21 | Present results of each meta-analysis done, including confidence intervals and measures of consistency. | 8-11 |
| Risk of bias across  studies | 22 | Present results of any assessment of risk of bias across studies (see Item 15). | 10 |
| Additional analysis | 23 | Give results of additional analyses, if done (e.g., sensitivity or subgroup analyses, meta-regression [see Item 16]). | 8-10 |
| **DISCUSSION** |  |  |  |
| Summary of evidence | 24 | Summarize the main findings including the strength of evidence for each main outcome; consider their relevance to key groups (e.g., healthcare providers, users, and policy makers). | 10-12 |
| Limitations | 25 | Discuss limitations at study and outcome level (e.g., risk of bias), and at review-level (e.g., incomplete retrieval of identified research, reporting bias). | 12 |
| Conclusions | 26 | Provide a general interpretation of the results in the context of other evidence, and implications for future research. | 12-13 |
| **FUNDING** |  |  |  |
| Funding | 27 | Describe sources of funding for the systematic review and other support (e.g., supply of data); role of funders for the systematic review. | 14 |

**Table S2. Operational definitions of primary variables.**

| **Variable** | **Definition** |
| --- | --- |
| Cognitive function | Cognitive function is a broad construct encompassing key domains such as memory, executive function, attention, and processing speed. In this study, we extracted standardized mean differences (Hedges’ g) for individual cognitive outcomes as reported in the included studies. When available, multiple domain-specific scores were included to reflect a comprehensive representation of cognitive function. This multidimensional approach is consistent with previous meta-analyses [9], allowing for a more accurate estimation of the overall cognitive effects of physical exercise interventions. |
| Physical exercise | Physical exercise defined as a structured, repetitive form of physical activity designed to improve or maintain physical fitness [15]. |
| Brain volume | Brain volume was operationalized as a structural neuroimaging outcome reflecting the size of cerebral tissue, including both gray matter and white matter. In accordance with prior meta-analyses [4], we included whole brain volume, regional brain volumes (e.g., hippocampus, frontal cortex), and gray/white matter volumes, recognizing that each reflects different but complementary aspects of neuroplasticity. Effect sizes were computed using standardized mean differences (Hedges’ g), and where multiple brain regions were reported within a study, these were treated as nested effects within a multilevel meta-analytic model to account for statistical dependence. |
| Exercise-induced physical adaptations | Exercise-induced physical adaptations refer to measurable improvements in physiological and functional capacities resulting from structured physical exercise interventions. In this study, we focused on three core domains: muscle function (e.g., strength, endurance), motor function (e.g., balance, coordination, mobility), and cardiorespiratory fitness (e.g., VO₂max, heart rate recovery). These adaptations were assessed using standardized performance-based measures or physiological indices as reported in the included studies. |

**Table S3. Descriptions of moderators.**

| **Subgroup** | **Level** | **Definition** |
| --- | --- | --- |
| Exercise frequency | High frequency | Exercise programs with three or more sessions per week. |
| Exercise frequency | Low frequency | Exercise programs with fewer than three sessions per week. |
| Exercise intensity^1^ | Light intensity | Light intensity includes gentle aerobic movements that do not noticeably alter the breathing rate and can be comfortably maintained for over 60 minutes [16]. |
| Exercise intensity^1^ | Moderate intensity | Moderate intensity involves an aerobic effort that allows uninterrupted conversation and can generally be sustained for 30 to 60 minutes [16]. |
| Exercise intensity^1^ | Vigorous intensity | Vigorous intensity leads to a significant increase in breathing, making it difficult to sustain conversation without interruption, and is typically sustainable for up to 30 minutes [16]. |
| Exercise intensity^1^ | High intensity | High intensity requires maximal exertion, generally unsustainable for more than about 10 minutes [16]. |
| Assessment timepoint | Follow-up | The timepoint at which assessments are conducted some time after the intervention has ended |
| Assessment timepoint | Post-intervention | The timepoint immediately following the completion of the intervention |
| Assessment timepoint | Mid-intervention | The timepoint during the intervention period |
| Clinical status | Clinical cohorts | Participants explicitly diagnosed with neurocognitive or neurological conditions, such as Alzheimer’s disease, mild cognitive impairment, Parkinson’s disease, stroke, or other relevant clinical syndromes, as defined by the original studies. |
| Clinical status | Non-clinical cohorts | Participants without reported diagnoses of neurological or psychiatric disorders, including healthy older adults, cognitively normal individuals, and general populations such as children, adolescents, and younger adults. |

**Notes:** ^1^If the studies did not provide objective or subjective measures of exercise intensity, two researchers extracted the corresponding metabolic equivalent of Task (METs) using descriptions from included studies and classified intensity according to the Compendium of Physical Activities.

## Table S4. Reference list of included studies.

| **Number** | **Full reference** |
| --- | --- |
| 1 | Bergman F, Matsson-Frost T, Jonasson L, Ekblom Ö. Walking time is associated with hippocampal volume in overweight and obese office workers. Front Hum Neurosci. 2020;14:307. doi:10.3389/fnhum.2020.00307. |
| 2 | Best JR, Chiu BK, Liang Hsu C, Nagamatsu LS, Liu-Ambrose T. Long-term effects of resistance exercise training on cognition and brain volume in older women: Results from a randomized controlled trial. J Int Neuropsychol Soc. 2015;21(9):745-756. doi:10.1017/S1355617715000673. |
| 3 | Blackmore DG, Schaumberg MA, Ziaei M, Rietze RL, Bartlett PF. Long-term improvement in hippocampal-dependent learning ability in healthy, aged individuals following high-intensity interval training. Aging Dis. 2024. doi:10.14336/AD.2024.0642. |
| 4 | Boecker H, Daamen M, Kunz L, Kuhn T, Merz CJ. Hippocampal subfield plasticity is associated with improved spatial memory. Commun Biol. 2024;7(1):271. doi:10.1038/s42003-024-05949-5. |
| 5 | Broadhouse KM, Singh MF, Suo C, Gates N, Valenzuela MJ. Hippocampal plasticity underpins long-term cognitive gains from resistance exercise in MCI. Neuroimage Clin. 2020;25:102182. doi:10.1016/j.nicl.2020.102182. |
| 6 | Callisaya ML, Daly RM, Sharman JE, Bruce DG. Feasibility of a multi-modal exercise program on cognition in older adults with type 2 diabetes: A pilot randomized controlled trial. BMC Geriatr. 2017;17(1):237. doi:10.1186/s12877-017-0635-9. |
| 7 | Castells-Sánchez A, Roig-Coll F, Dacosta-Aguayo R, Jurado MA. Molecular and brain volume changes following aerobic exercise, cognitive and combined training in physically inactive healthy late-middle-aged adults: The Projecte Moviment randomized controlled trial. Front Hum Neurosci. 2022;16:854175. doi:10.3389/fnhum.2022.854175. |
| 8 | Damme KSF, Gupta T, Ristanovic I, Wood SJ. Exercise intervention in individuals at clinical high risk for psychosis: Benefits to fitness, symptoms, hippocampal volumes, and functional connectivity. Schizophr Bull. 2022;48(6):1394-1405. doi:10.1093/schbul/sbac084. |
| 9 | Demnitz N, Stathi A, Withall J, Greig CA. Hippocampal maintenance after a 12-month physical activity intervention in older adults: The REACT MRI study. Neuroimage Clin. 2022;35:102762. doi:10.1016/j.nicl.2021.102762. |
| 10 | Erickson KI, Voss MW, Prakash RS, Chaddock L, Kramer AF. Exercise training increases size of hippocampus and improves memory. Proc Natl Acad Sci U S A. 2011;108(7):3017-3022. doi:10.1073/pnas.1015956108. |
| 11 | Ehlers DK, Daugherty AM, Burzynska AZ, Kramer AF. Regional brain volumes moderate, but do not mediate, the effects of group-based exercise training on reductions in loneliness in older adults. Front Aging Neurosci. 2017;9:110. doi:10.3389/fnagi.2017.00110. |
| 12 | Feys P, Moumdjian L, Van Halewyck F, Feys L. Effects of an individual 12-week community-located “start-to-run” program on physical capacity, walking, fatigue, cognitive function, brain volumes, and structures in persons with multiple sclerosis. Mult Scler. 2019;25(1):92-103. doi:10.1177/1352458517740211. |
| 13 | Frederiksen KS, Larsen CT, Hasselbalch SG, Christensen H. A 16-week aerobic exercise intervention does not affect hippocampal volume and cortical thickness in mild to moderate Alzheimer’s disease. Front Aging Neurosci. 2018;10:293. doi:10.3389/fnagi.2018.00293. |
| 14 | Frodl T, Strehl K, Carballedo A, Scheuerecker J. Aerobic exercise increases hippocampal subfield volumes in younger adults and prevents volume decline in the elderly. Brain Imaging Behav. 2020;14(4):1577-1587. doi:10.1007/s11682-019-00088-6. |
| 15 | Frost NJ, Weinborn M, Gignac GE, Bucks RS. The effect of self-paced exercise intensity and cardiorespiratory fitness on frontal grey matter volume in cognitively normal older adults: A randomized controlled trial. J Int Neuropsychol Soc. 2022;28(9):902-915. doi:10.1017/S1355617721001132. |
| 16 | Guzman J, Aguiñaga S, Balbim GM, Marquez DX. The effects of the BAILAMOS dance program on hippocampal volume in older Latinos: A randomized controlled pilot study. Transl Behav Med. 2021;11:1857-1862. doi:10.1093/tbm/ibab009. |
| 17 | Gylling AT, Eriksen CS, Garde E, Mortensen EL. The influence of prolonged strength training upon muscle and fat in healthy and chronically diseased older adults. Exp Gerontol. 2020;136:110939. doi:10.1016/j.exger.2020.110939. |
| 18 | Jonasson LS, Nyberg L, Kramer AF, Adolfsson R. Aerobic exercise intervention, cognitive performance, and brain structure: Results from the physical influences on brain in aging (PHIBRA) study. Front Aging Neurosci. 2017;8:336. |
| 19 | Kaiser A, Reneman L, Solleveld MM, Schutter DJ. A randomized controlled trial on the effects of a 12-week high- vs. low-intensity exercise intervention on hippocampal structure and function in healthy, young adults. Front Psychiatry. 2022;12:780095. doi:10.3389/fpsyt.2021.780095. |
| 20 | Krogh J, Rostrup E, Thomsen C, Nordentoft M. The effect of exercise on hippocampal volume and neurotrophins in patients with major depression: A randomized clinical trial. J Affect Disord. 2014;165:24-30. doi:10.1016/j.jad.2014.04.041. |
| 21 | Koevoets EW, Geerlings MI, Monninkhof EM, Peeters PH. Effect of physical exercise on the hippocampus and global grey matter volume in breast cancer patients: A randomized controlled trial (PAM study). Neuroimage Clin. 2023;37:103292. doi:10.1016/j.nicl.2022.103292. |
| 22 | Lenze EJ, Voegtle M, Miller JP, Warren SL. Effects of mindfulness training and exercise on cognitive function in older adults: A randomized clinical trial. JAMA. 2022;328:2218-2229. doi:10.1001/jama.2022.21680. |
| 23 | Lin J, Chan SK, Lee EH, Hui CL. Aerobic exercise and yoga improve neurocognitive function in women with early psychosis. NPJ Schizophr. 2015;1:15047. doi:10.1038/npjschz.2015.47. |
| 24 | Liu J, Chen L, Chen X, Yang Y. Modulatory effects of different exercise modalities on the functional connectivity of the periaqueductal grey and ventral tegmental area in patients with knee osteoarthritis: A randomised multimodal magnetic resonance imaging study. Br J Anaesth. 2019;123:506-518. doi:10.1016/j.bja.2019.06.017. |
| 25 | Liu J, Tao J, Xia R, Liu W. Mind-body exercise modulates locus coeruleus and ventral tegmental area functional connectivity in individuals with mild cognitive impairment. Front Aging Neurosci. 2021;13:646807. doi:10.3389/fnagi.2021.646807. |
| 26 | Liu-Ambrose T, Best JR, Davis JC, Hsu C. Aerobic exercise and vascular cognitive impairment: A randomized controlled trial. Neurology. 2016;87:2082-2090. |
| 27 | Langeskov-Christensen M, Grøndahl Hvid L, Nygaard MKE, Madsen TH. Efficacy of high-intensity aerobic exercise on brain MRI measures in multiple sclerosis. Neurology. 2021;96:e213. doi:10.1212/WNL.0000000000011241. |
| 28 | Maurer A, Klein J, Claus J, Fischer J. Effects of a 6-month aerobic exercise intervention on mood and amygdala functional plasticity in young untrained subjects. Int J Environ Res Public Health. 2022;19:6078. doi:10.3390/ijerph19106078. |
| 29 | Morris JK, Vidoni ED, Johnson DK, Burns JM. Aerobic exercise for Alzheimer's disease: A randomized controlled pilot trial. PLoS One. 2017;12:e0170547. doi:10.1371/journal.pone.0170547. |
| 30 | Mortimer JA, Ding D, Borenstein AR, Wu Y. Changes in brain volume and cognition in a randomized trial of exercise and social interaction in a community-based sample of non-demented Chinese elders. J Alzheimers Dis. 2012;30:757-766. |
| 31 | Malchow B, Keeser D, Keller K, Hasan A. Effects of endurance training on brain structures in chronic schizophrenia patients and healthy controls. Schizophr Res. 2016;173:182-191. doi:10.1016/j.schres.2015.01.005. |
| 32 | Matura S, Fleckenstein J, Deichmann R, Pantel J. Effects of aerobic exercise on brain metabolism and grey matter volume in older adults: Results of the randomized controlled SMART trial. Transl Psychiatry. 2017;7:e135. doi:10.1038/tp.2017.135. |
| 33 | Nagamatsu LS, Weinstein AM, Erickson KI, Liu-Ambrose T. Exercise mode moderates the relationship between mobility and basal ganglia volume in healthy older adults. J Am Geriatr Soc. 2016;64:102-108. |
| 34 | Niemann C, Godde B, Voelcker-Rehage C. Not only cardiovascular, but also coordinative exercise increases hippocampal volume in older adults. Front Aging Neurosci. 2014;6:170. |
| 35 | Niemann C, Godde B, Staudinger UM, Voelcker-Rehage C. Exercise-induced changes in basal ganglia volume and cognition in older adults. Neuroscience. 2014;281:147-163. |
| 36 | Ortega FB, Mora-Gonzalez J, Cadenas-Sanchez C, Catena A. Effects of an exercise program on brain health outcomes for children with overweight or obesity: The ActiveBrains randomized clinical trial. JAMA Netw Open. 2022;5:e2227893. doi:10.1001/jamanetworkopen.2022.27893. |
| 37 | Pajonk FG, Wobrock T, Gruber O, Falkai P. Hippocampal plasticity in response to exercise in schizophrenia. Arch Gen Psychiatry. 2010;67:133-143. |
| 38 | Pani J, Reitlo LS, Evensmoen HR, Haberg AK. Effect of 5 years of exercise intervention at different intensities on brain structure in older adults from the general population: A Generation 100 substudy. Clin Interv Aging. 2021;16:1485-1501. doi:10.2147/CIA.S318679. |
| 39 | Riemenschneider M, Hvid LG, Ringgaard S, Dalgas U. Investigating the potential disease-modifying and neuroprotective efficacy of exercise therapy early in the disease course of multiple sclerosis: The Early Multiple Sclerosis Exercise Study (EMSES). Mult Scler. 2022;28:1620-1629. doi:10.1177/13524585221079200. |
| 40 | Rocca MA, Valsasina P, Romanò F, Filippi M. Cognitive rehabilitation effects on grey matter volume and Go-NoGo activity in progressive multiple sclerosis: Results from the CogEx trial. J Neurol Neurosurg Psychiatry. 2024. doi:10.1136/jnnp-2024-333460. |
| 41 | Rosano C, Guralnik J, Pahor M, Anton SD. Hippocampal response to a 24-month physical activity intervention in sedentary older adults. Am J Geriatr Psychiatry. 2017;25:209-217. doi:10.1016/j.jagp.2016.11.007. |
| 42 | Savšek L, Stergar T, Strojnik V, Hadžić V. Impact of aerobic exercise on clinical and magnetic resonance imaging biomarkers in persons with multiple sclerosis: An exploratory randomized controlled trial. J Rehabil Med. 2021;53. doi:10.2340/16501977-2814. |
| 43 | Scheewe TW, van Haren NE, Sarkisyan G, Kahn RS. Exercise therapy, cardiorespiratory fitness and their effect on brain volumes: A randomised controlled trial in patients with schizophrenia and healthy controls. Eur Neuropsychopharmacol. 2013;23:675-685. |
| 44 | Sexton CE, Betts JF, Dennis A, Filippini N. The effects of an aerobic training intervention on cognition, grey matter volumes and white matter microstructure. Physiol Behav. 2020;223:112923. doi:10.1016/j.physbeh.2020.112923. |
| 45 | Soshi T, Andersson M, Kawagoe T, Nishikawa K. Prefrontal plasticity after a 3-month exercise intervention in older adults relates to enhanced cognitive performance. Cereb Cortex. 2021;31:4501-4517. doi:10.1093/cercor/bhab102. |
| 46 | Suo C, Singh MF, Gates N, Valenzuela MJ. Therapeutically relevant structural and functional mechanisms triggered by physical and cognitive exercise. Mol Psychiatry. 2016;21:1645. doi:10.1038/mp.2016.57. |
| 47 | Tarumi T, Patel NR, Tomoto T, Zhang R. Aerobic exercise training and neurocognitive function in cognitively normal older adults: A one-year randomized controlled trial. J Intern Med. 2022;292:788-803. doi:10.1111/joim.13534. |
| 48 | Tarumi T, Thomas BP, Tseng BY, Zhang R. Cerebral white matter integrity in amnestic mild cognitive impairment: A 1-year randomized controlled trial of aerobic exercise training. J Alzheimers Dis. 2020;73:489-501. doi:10.3233/JAD-190875. |
| 49 | Tarumi T, Rossetti H, Thomas BP, Cullum CM. Exercise training in amnestic mild cognitive impairment: A one-year randomized controlled trial. J Alzheimers Dis. 2019;71:421-433. doi:10.3233/JAD-181175. |
| 50 | ten Brinke LF, Bolandzadeh N, Nagamatsu LS, Liu-Ambrose T. Aerobic exercise increases hippocampal volume in older women with probable mild cognitive impairment: A 6-month randomised controlled trial. Br J Sports Med. 2015;49:248-254. doi:10.1136/bjsports-2013-093184. |
| 51 | Venkatraman VK, Sanderson A, Cox KL, Ellis KA. Effect of a 24-month physical activity program on brain changes in older adults at risk of Alzheimer's disease: The AIBL active trial. Neurobiol Aging. 2020;89:132-141. |
| 52 | Vidoni ED, Morris JK, Watts A, Burns JM. Effect of aerobic exercise on amyloid accumulation in preclinical Alzheimer's: A 1-year randomized controlled trial. PLoS One. 2021;16:e0244893. doi:10.1371/journal.pone.0244893. |
| 53 | Vints WAJ, Šeikinaitė J, Gökçe E, Vandenberghe R. Resistance exercise effects on hippocampus subfield volumes and biomarkers of neuroplasticity and neuroinflammation in older adults with low and high risk of mild cognitive impairment: A randomized controlled trial. GeroScience. 2024;46:3971-3991. doi:10.1007/s11357-024-01110-6. |
| 54 | Wang Y, Wang L, Yan J, Chen F. Aerobic training increases hippocampal volume and protects cognitive function for type 2 diabetes patients with normal cognition. Exp Clin Endocrinol Diabetes. 2023;131:605-614. doi:10.1055/a-2105-0799. |
| 55 | Wender CLA, Sandroff BM, Krch D, Motl RW. The preliminary effects of moderate aerobic training on cognitive function in people with TBI and significant memory impairment: A proof-of-concept randomized controlled trial. Neurocase. 2021;27:430-435. doi:10.1080/13554794.2021.1990964. |
| 56 | Woodward ML, Lin J, Gicas KM, Thornton AE. Medial temporal lobe cortical changes in response to exercise interventions in people with early psychosis: A randomized controlled trial. Schizophr Res. 2020;223:87-95. |
| 57 | Wan M, Xia R, Lin H, Tao J. Baduanjin exercise modulates the hippocampal subregion structure in community-dwelling older adults with cognitive frailty. Front Aging Neurosci. 2022;14:956273. doi:10.3389/fnagi.2022.956273.[https://doi.org/10.3389/fnagi.2022.956273](https://doi.org/10.3389/fnagi.2022.956273) |
| 58 | Yu F, Mathiason MA, Han S, Sui X. Mechanistic effects of aerobic exercise in Alzheimer's disease: Imaging findings from the pilot FIT-AD trial. Front Aging Neurosci. 2021;13:703691. doi:10.3389/fnagi.2021.703691. |
| 59 | Zhu L, Yu Q, Herold F, Müller NG. Brain structure, cardiorespiratory fitness, and executive control changes after a 9-week exercise intervention in young adults: A randomized controlled trial. Life. 2021;11:292. doi:10.3390/life11040292. |

## Table S5. Reasons for study exclusion.

**A. Not a randomized controlled trial**

Bunketorp Käll L, Malmgren H, Olsson E, Lindén T, Nilsson M. Effects of a curricular physical activity intervention on children's school performance, wellness, and brain development. J Sch Health. 2015;85(10):704-713. doi:10.1111/josh.12303.

Cui X, Gui W, Miao J, Liu X, Zhu X, Zheng Z, et al. A combined intervention of aerobic exercise and video game in older adults: The efficacy and neural basis on improving mnemonic discrimination. J Gerontol A Biol Sci Med Sci. 2023;78(8):1436-1444.

Fissler P, Müller HP, Küster OC, Laptinskaya D, Thurm F, Woll A, et al. No evidence that short-term cognitive or physical training programs or lifestyles are related to changes in white matter integrity in older adults at risk of dementia. Front Hum Neurosci. 2017;11:239960.

Kjølhede T, Siemonsen S, Wenzel D, Stellmann JP, Ringgaard S, Pedersen BG, et al. Can resistance training impact MRI outcomes in relapsing-remitting multiple sclerosis? Mult Scler J. 2018;24(10):1356-1365.

Parker BA, Thompson PD, Jordan KC, Grimaldi AS, Assaf M, Jagannathan K, Pearlson GD. Effect of exercise training on hippocampal volume in humans: A pilot study. Res Q Exerc Sport. 2011;82(3):585-591.

Satoh M, Ogawa JI, Tokita T, Nakaguchi N, Nakao K, Kida H, Tomimoto H. The effects of physical exercise with music on cognitive function of elderly people: Mihama-Kiho project. PLoS One. 2014;9(4):e95230. doi:10.1371/journal.pone.0095230.

Szulc-Lerch KU, Timmons BW, Bouffet E, Laughlin S, de Medeiros CB, Skocic J, et al. Repairing the brain with physical exercise: cortical thickness and brain volume increases in long-term pediatric brain tumor survivors in response to a structured exercise intervention. Neuroimage Clin. 2018;18:972-985.

Tabei KI, Satoh M, Ogawa JI, Tokita T, Nakaguchi N, Nakao K, et al. Physical exercise with music reduces gray and white matter loss in the frontal cortex of elderly people: the Mihama-Kiho scan project. Front Aging Neurosci. 2017;9:174.

Teixeira CVL, de Rezende TJR, Weiler M, Magalhães TN C, Carletti-Cassani AFMK, Silva TQAC, et al. Cognitive and structural cerebral changes in amnestic mild cognitive impairment due to Alzheimer's disease after multicomponent training. Alzheimers Dement (Transl Res Clin Interv). 2018;4:473-480.

Thomas AG, Dennis A, Rawlings NB, Stagg CJ, Matthews L, Morris M, et al. Multi-modal characterization of rapid anterior hippocampal volume increase associated with aerobic exercise. Neuroimage. 2016;131:162-170.

Vaquero L, Rodríguez‐Fornells A, Pera‐Jambrina MÁ, Bruna J, Simó M. Plasticity in bilateral hippocampi after a 3‐month physical activity programme in lung cancer patients. Eur J Neurol. 2021;28(4):1324-1333.

Wagner G, Herbsleb M, Cruz FDL, Schumann A, Brünner F, Schachtzabel C, et al. Hippocampal structure, metabolism, and inflammatory response after a 6-week intense aerobic exercise in healthy young adults: a controlled trial. J Cereb Blood Flow Metab. 2015;35(10):1570-1578.

Maaß A, Düzel S, Goerke M, Becke A, Sobieray U, Neumann K, et al. Vascular hippocampal plasticity after aerobic exercise in older adults. Mol Psychiatry. 2015;20(5):585-593.

Maass A, Düzel S, Brigadski T, Goerke M, Becke A, Sobieray U, et al. Relationships of peripheral IGF-1, VEGF and BDNF levels to exercise-related changes in memory, hippocampal perfusion and volumes in older adults. Neuroimage. 2016;131:142-154.

**B. No brain volume outcome**

Bolandzadeh N, Tam R, Handy TC, Nagamatsu LS, Hsu CL, Davis JC, et al. Resistance training and white matter lesion progression in older women: exploratory analysis of a 12‐month randomized controlled trial. J Am Geriatr Soc. 2015;63(10):2052-2060.

Crockett RA, Hsu CL, Dao E, Tam R, Eng JJ, Handy TC, Liu-Ambrose T. Weight for it: resistance training mitigates white matter hyperintensity-related disruption to functional networks in older females. J Alzheimers Dis. 2022;90(2):553-563.

Dao E, Barha CK, Best JR, Hsiung GY, Tam R, Liu-Ambrose T. The effect of aerobic exercise on white matter hyperintensity progression may vary by sex. Can J Aging. 2019;38(2):236-244.

Oh J, Crockett RA, Hsu CL, Dao E, Tam R, Liu-Ambrose T. Resistance training maintains white matter and physical function in older women with cerebral small vessel disease: an exploratory analysis of a randomized controlled trial. J Alzheimers Dis Rep. 2023;7(1):627-639.

Pani J, Marzi C, Stensvold D, Wisløff U, Håberg AK, Diciotti S. Longitudinal study of the effect of a 5-year exercise intervention on structural brain complexity in older adults. A Generation 100 substudy. Neuroimage. 2022;256:119226. doi:10.1016/j.neuroimage.2022.119226.

Rektorova I, Klobusiakova P, Balazova Z, Kropacova S, Sejnoha Minsterova A, Grmela R, et al. Brain structure changes in nondemented seniors after six‐month dance‐exercise intervention. Acta Neurol Scand. 2020;141(1):90-97.

Ruscheweyh R, Willemer C, Krüger K, Duning T, Warnecke T, Sommer J, et al. Physical activity and memory functions: an interventional study. Neurobiol Aging. 2011;32(7):1304-1319.

Suzuki T, Shimada H, Makizako H, Doi T, Yoshida D, Ito K, et al. A randomized controlled trial of multicomponent exercise in older adults with mild cognitive impairment. PLoS One. 2013;8(4):e61483. doi:10.1371/journal.pone.0061483.

**C. Insufficient information**

de Zoete RM, McMahon KL, Coombes JS, Sterling M. The effects of physical exercise on structural, functional, and biochemical brain characteristics in individuals with chronic whiplash‐associated disorder: A pilot randomized clinical trial. Pain Pract. 2023;23(7):759-775.

Scharf C, Koschutnig K, Zussner T, Fink A, Tilp M. Twelve weeks of physical exercise breaks with coordinative exercises at the workplace increase the sulcal depth and decrease gray matter volume in brain structures related to visuomotor processes. Brain Struct Funct. 2024;229(1):63-74.

Martin-Willett R, Ellingson JE, Fries J, Helmuth T, Karoly H, Giordano G, et al. Few structural brain changes associated with moderate-intensity interval training and low-intensity continuous training in a randomized trial of fitness and older adults. J Aging Phys Act. 2020;29(3):505-515.

**D. Ineligible a control group**

Dean DJ, Bryan AD, Newberry R, Gupta T, Carol E, Mittal VA. A supervised exercise intervention for youth at risk for psychosis: an open-label pilot study. J Clin Psychiatry. 2017;78(9):6918.

van Aalst J, Jennen L, Demyttenaere K, Sunaert S, Koole M, Ceccarini J, et al. Twelve-week yoga vs. aerobic cycling initiation in sedentary healthy subjects: a behavioral and multiparametric interventional PET/MR study. Front Psychiatry. 2021;12:739356.

Kleemeyer MM, Kühn S, Prindle J, Bodammer NC, Brechtel L, Garthe A, et al. Changes in fitness are associated with changes in hippocampal microstructure and hippocampal volume among older adults. Neuroimage. 2016;131:155-161.

Sandroff BM, Wylie GR, Baird JF, Jones CD, Diggs MD, Genova H, et al. Effects of walking exercise training on learning and memory and hippocampal neuroimaging outcomes in MS: a targeted, pilot randomized controlled trial. Contemp Clin Trials. 2021;110:106563.

Yang H, Leaver AM, Siddarth P, Paholpak P, Ercoli L, St Cyr NM, et al. Neurochemical and neuroanatomical plasticity following memory training and yoga interventions in older adults with mild cognitive impairment. Front Aging Neurosci. 2016;8:277.

Müller P, Rehfeld K, Schmicker M, Hökelmann A, Dordevic M, Lessmann V, et al. Evolution of neuroplasticity in response to physical activity in old age: the case for dancing. Front Aging Neurosci. 2017;9:56.

**E. Ineligible intervention group**

Espeland MA, Erickson K, Neiberg RH, Jakicic JM, Wadden TA, Wing RR, et al. Brain and white matter hyperintensity volumes after 10 years of random assignment to lifestyle intervention. Diabetes Care. 2016;39(5):764-771.

Khonsari NM, Badrfam R, Mohammdi MR, Rastad H, Etemadi F, Vafaei Z, Zandifar A. Effect of aerobic exercise as adjunct therapy on the improvement of negative symptoms and cognitive impairment in patients with schizophrenia: a randomized, case-control clinical trial. J Psychosoc Nurs Ment Health Serv. 2022;60(5):38-43.

McEwen SC, Jarrahi B, Ventura J, Subotnik KL, Nguyen J, Woo SM, Nuechterlein KH. A combined exercise and cognitive training intervention induces fronto-cingulate cortical plasticity in first-episode psychosis patients. Schizophr Res. 2023;251:12-21.

Head D, Singh T, Bugg JM. The moderating role of exercise on stress-related effects on the hippocampus and memory in later adulthood. Neuropsychology. 2012;26(2):133.

Smith JC, Nielson KA, Woodard JL, Seidenberg M, Durgerian S, Hazlett KE, et al. Physical activity reduces hippocampal atrophy in elders at genetic risk for Alzheimer's disease. Front Aging Neurosci. 2014;6:61.

Um YH, Wang SM, Kim NY, Kang DW, Na HR, Lee CU, Lim HK. Effects of moderate intensity exercise on the cortical thickness and subcortical volumes of preclinical Alzheimer’s disease patients: A pilot study. Psychiatry Investig. 2020;17(6):613.

## Table S6. Information of included studies.

| Author & year | Population | Age | Gender  (female) | Intervention group(s) | Control group(s) | Time points | MRI acquisition | Analysis method |
| --- | --- | --- | --- | --- | --- | --- | --- | --- |
| Best et al., 2015 | Non-clinical cohorts | 69±2 | 100% | 1. RES 52 × 120 min/w, MI (n=29) 2. RES 52 × 60 min/w, MI (n=29) | Active (n=18) | Pre (0 w)  Post (48 w)  Follow-up (96 w) | 3T Siemens MRI  3D MPRAGE  1 mm³ isotropic | VBM |
| Boecker et al., 2024 | Clinical cohorts | 23±4 | 64% | AER 48 × 35 min/w, VI (n=17) | Passive (n=10) | Pre (0 w)  Mid (8 w)  Mid (24 w)  Post (48 w) | 3T Siemens Prisma  3D MPRAGE  0.9 mm³ isotropic voxels | FreeSurfer |
| Broadhouse et al., 2020 | Clinical cohorts | 69±6 | 69% | RES 26 × 225 min/w, VI (n=27) | Active (n=17) | Pre (0 w)  Post (24 w)  Follow-up (72 w) | 3T Siemens Skyra MRI scanner  3D MPRAGE  1.0 mm³ isotropic | FreeSurfer |
| Blackmore et al., 2024 | Non-clinical cohorts | 71±1 | 53% | 1. AER 24 × 80 min/w, VI (n=70) 2. AER 24 × 90 min/w, MI (n=72) | Active (n=72) | Pre (0 w)  Post (24 w)  Follow-up (48 w) | 3T Siemens Skyra  3D MPRAGE  1.0 mm³ isotropic voxels | FreeSurfer |
| Bergman et al., 2020 | Non-clinical cohorts | 51±6 | 55% | AER 52 × 240 min/w, LI (n=40) | Passive (n=40) | Pre (0 w)  Post (52 w) | 3T Siemens Trio scanner  3D MPRAGE  1 mm isotropic voxels | FreeSurfer |
| Callisaya et al., 2017 | Non-clinical cohorts | 66±4 | 48% | MCE 24 × 135 min/w, VI (n=26) | Active (n=24) | Pre (0 w)  Post (24 w) | 3T Siemens Magnetom Trio  3D MPRAGE  1 mm³ isotropic | FreeSurfer |
| Castells-Sánchez et al., 2022 | Non-clinical cohorts | 58±5 | 62% | AER 12 × 187 min/w, MI (n=33) | Passive (n=15) | Pre (0 w)  Post (12 w) | 3T Siemens MAGNETOM Trio  3D MPRAGE  1 mm³ isotropic voxel | FreeSurfer |
| Demnitz et al., 2022 | Non-clinical cohorts | 76±6 | 62% | MCE 48 × 90 min/w, MI (n=54) | Active (n=48) | Pre (0 w)  Mid (24 w)  Post (48 w) | 3T Siemens MAGNETOM Trio  3D MPRAGE  1 mm³ isotropic | FreeSurfer |
| Damme et al., 2022 | Non-clinical cohorts | 21±2 | 53% | AER 12 × 60 min/w, VI (n = 18) | Passive (n=20) | Pre (0 w)  Post (12 w) | 3T Siemens Prisma  3D MPRAGE  1 mm³ isotropic voxel | FreeSurfer |
| Erickson et al., 2011 | Non-clinical cohorts | 66±5 | 73% | AER 48 × 40 min/w, MI (n=60) | Active (n=60) | Pre (0 w)  Mid (24 w)  Post (48 w) | 3T scanner NR  3D MPRAGE  1.3 mm³ isotropic voxel | FSL |
| Ehlers et al., 2017 | Non-clinical cohorts | 65±4 | 68% | 1. AER 24 × 180 min/w,MI (n=69) 2. AER 24 × 180 min/w,MI (n=54) | Active (n=70) | Pre (0 w)  Post (24 w) | 3T Siemens TIM Trio scanner  3D MPRAGE  1 mm³ isotropic voxel | FreeSurfer |
| Frost et al., 2022 | Non-clinical cohorts | 69±5 | 55% | 1. AER 24 × 100 min/w, VI (n=33) 2. AER 24 × 100 min/w, LI (n=34) | Active (n=31) | Pre (0 w)  Post (24 w)  Follow-up (72 w) | 3T Siemens Prisma  3D MPRAGE  1 mm³ isotropic voxel | FreeSurfer |
| Frederiksen et al., 2018 | Clinical cohorts | 68±7 | 29% | AER 16 × 180 min/w, VI (n=36) | Passive (n=35) | Pre (0 w)  Post (16 w) | 3T Siemens Trio  3D MPRAGE  1 mm³ isotropic voxel | FreeSurfer |
| Frodl et al., 2020 | Non-clinical cohorts | 44±13 | 66% | AER 16 × 85 min/w, MI (n=20) | Passive (n=19) | Pre (0 w)  Post (16 w) | 3T Siemens Prisma  3D MPRAGE  1 mm³ isotropic voxel | FreeSurfer |
| Feys et al., 2019 | Clinical cohorts | 40±9 | 90% | AER 12 × 135 min/w, MI (n=21) | Passive (n=21) | Pre (0 w)  Post (12 w) | 3T Philips Achieva  3D MPRAGE  1 mm³ isotropic voxel | FreeSurfer |
| Gylling et al., 2024 | Non-clinical cohorts | 66±2 | 60% | 1. RES 48 × NR min/w, VI (n=149) 2. RES 48 × NR min/w, MI (n=154) | Active (n=148) | Pre (0 w)  Post (48 w) | 3T Siemens Skyra  3D MPRAGE  1 mm³ isotropic voxel | FreeSurfer |
| Guzman et al., 2021 | Non-clinical cohorts | 67±6 | 72% | AER 16 × 120 min/w, MI (n=12) | Passive (n=12) | Pre (0 w)  Post (16 w) | 3T GE Discovery MR750  3D BRAVO  0.42×0.42×1.5 mm³ voxel | FreeSurfer |
| Jonasson et al., 2017 | Non-clinical cohorts | 68±2 | 80% | AER 24 × 135 min/w, MI (n=29) | Active (n=29) | Pre (0 w)  Post (24 w) | 3T GE Discovery 750  3D BRAVO  1 mm³ isotropic voxel | FreeSurfer |
| Kaiser et al., 2022 | Non-clinical cohorts | 23±3 | 53% | AER 12 × 135 min/w, VI (n=26) | Active (n=26) | Pre (0 w)  Post (12 w) | 3T Siemens Skyra  3D MPRAGE  1 mm³ isotropic voxel | FreeSurfer |
| Krogh et al., 2014 | Clinical cohorts | 41±12 | 67% | AER 12 × 135min/w, VI (n=41) | Active (n=38) | Pre (0 w)  Post (12 w) | 3T Philips Achieva  3D MPRAGE  1 mm³ isotropic voxel | FreeSurfer |
| Koevoets et al., 2023 | Clinical cohorts | 52±8 | 100% | MCE 24 × 240 min/w, MI (n=91) | Passive (n=90) | Pre (0 w)  Post (24 w) | 3T Siemens Skyra  3D MPRAGE  1 mm³ isotropic voxel | FreeSurfer |
| Lin et al., 2015 | Clinical cohorts | 24±7 | 100% | 1. MBE 12 × 180 min/w, LI (n=45) 2. AER 12 × 180 min/w, LI (n=40) | Passive (n=39) | Pre (0 w)  Post (12 w) | 3T Philips Achieva  3D (sequence NR)  0.42×0.42×1.5 mm³ voxel | FreeSurfer |
| Lenze et al., 2022 | Clinical cohorts | 71±4 | 78% | MCE 72 × 180 min/w, MI (n=138) | Active (n=153) | Pre (0 w)  Mid (24 w)  Post (72 w) | 3T Siemens Prisma  3D MPRAGE  1 mm³ isotropic voxel | NR |
| Liu et al., 2019 | Clinical cohorts | 59±7 | 75% | 1. MBE 12 × 300 min/w, MI (n=28) 2. AER 12 × 300 min/w, MI (n=27) | Active (n=24) | Pre (0 w)  Post (12 w) | 3T Siemens (model NR)  3D MPRAGE  1 mm³ isotropic voxel | NR |
| Liu-Ambrose et al., 2010 | Non-clinical cohorts | 69±2 | 100% | 1. RES 48 × 120 min/w, MI (n=52) 2. RES 48 × 60 min/w, MI (n=54) | Active (n=49) | Pre (0 w)  Mid (24 w)  Post (48 w) | 1.5T GE Signa  3D (sequence NR)  voxel size NR | NR |
| Liu et al., 2021 | Clinical cohorts | 65±4 | 68% | 1. AER 24 × 60 min/w, MI (n=17) 2. MBE 24 × 60 min/w, MI (n=20) | Passive (n=20) | Pre (0 w)  Post (24 w) | 3T Siemens Trio  3D MPRAGE  1 mm³ isotropic voxel | FreeSurfer |
| Langeskov-Christensen et al., 2021 | Clinical cohorts | 44±9 | 60% | AER 24 × 90 min/w, VI (n=43) | Passive (n=43) | Pre (0 w)  Post (24 w) | 3T Siemens Trio  3D MPRAGE  1 mm³ isotropic voxel | FreeSurfer |
| Mortimer et al., 2012 | Clinical cohorts | 67±5 | 64% | 1. MBE 40 × 150 min/W, LI (n=30) 2. AER 40 × 150 min/W, LI (n=30) | Passive (n=30) | Pre (0 w)  Post (40 w) | 1.5T GE (model NR)  3D SPGR  1×1×1.5 mm³ isotropic voxel | NR |
| Morris et al., 2017 | Clinical cohorts | 72±7 | 43% | AER 26 × 150 min/w, VI (n=39) | Active (n=37) | Pre (0 w)  Post (26 w) | 3T Siemens Skyra 3D MPRAGE 1 mm³ isotropic voxel | FreeSurfer |
| Maurer et al., 2022 | Non-clinical cohorts | 23±3 | 61% | AER 24 × 105 min/w, VI (n=18) | Passive (n=10) | Pre (0 w)  Mid (8 w)  Mid (16 w)  Post (24 w) | 3T Siemens Prisma 3D MPRAGE 1 mm³ isotropic voxel | FreeSurfer |
| Malchow et al., 2015 | Clinical cohorts | 35±13 | 30% | AER 12 × 90 min/w, MI (n=20) | Active (n=19) | Pre (0 w)  Post (12 w) | 3T Siemens Magnetom Trio 3D MPRAGE 1 mm³ isotropic voxel | FreeSurfer |
| Matura et al., 2017 | Non-clinical cohorts | 74±6 | 42% | AER 12 × 90 min/w, VI (n=29) | Passive (n=24) | Pre (0 w)  Post (12 w) | 3T Siemens Magnetom Trio 3D MPRAGE 1 mm³ isotropic voxel | FreeSurfer |
| Niemann et al., 2014a | Non-clinical cohorts | 68±2 | 63% | 1. AER 48 × 157 min/w, MI (n=17) 2. MCE 48 × 157 min/w, MI (n=16) | Active (n=13) | Pre (0 w)  Mid (24 w)  Post (48 w) | 3T Siemens Magnetom Trio 3D MPRAGE 1 mm³ isotropic voxel | FreeSurfer |
| Niemann et al., 2014b | Non-clinical cohorts | 68±2 | 63% | 1. AER 48 × 157 min/w, MI (n=17) 2. MCE 48 × 157 min/w, MI (n=16) | Active (n=13) | Pre (0 w)  Mid (24 w)  Post (48 w) | 3T Siemens Magnetom Trio 3D MPRAGE 1 mm³ isotropic voxel | FreeSurfer |
| Nagamatsu et al., 2016 | Non-clinical cohorts | 66±5 | 74% | AER 48 × 120 min/w, VI (n=54) | Active (n=47) | Pre (0 w)  Post (48 w) | 3T Siemens Allegra 3D MPRAGE  1.3 mm³ isotropic voxel | FSL |
| Ortega et al., 2022 | Non-clinical cohorts | 10±1 | 41% | MCE 20 × 270 min/w, VI (n=57) | Passive (n=52) | Pre (0 w)  Post (20 w) | 3T Siemens Prisma 3D MPRAGE 1 mm³ isotropic voxel | FreeSurfer |
| Pajonk et al., 2010 | Clinical cohorts | 35±9 | 0% | AER 12 × 90 min/w, VI (n=8) | Active (n=8) | Pre (0 w)  Post (12 w) | 3T Siemens Trio 3D MPRAGE 1 mm³ isotropic voxel | FreeSurfer |
| Pani et al., 2021 | Non-clinical cohorts | 72±2 | 49% | 1. AER 240 × 80 min/w, HI (n=33) 2. AER 240 × 100 min/w, VI (n=24) | Passive (n=48) | Pre (0 w)  Mid (48 w)  Mid (144 w)  Post (240 w) | 3T Siemens Skyra 3D MPRAGE 1 mm³ isotropic voxel | FreeSurfer |
| Rocca et al., 2024 | Clinical cohorts | 51±6 | 61% | AER 12 × 43 min/w, VI (n=20) | Active (n=22) | Pre (0 w)  Post (12 w) | 3T Siemens Skyra 3D MPRAGE 1 mm³ isotropic voxel | FreeSurfer |
| Rosano et al., 2017 | Non-clinical cohorts | 76±5 | 70% | AER 96 × NR min/w, MI (n=10) | Active (n=16) | Pre (0 w)  Post (96 w) | 7T Siemens Magnetom 3D MPRAGE 0.7 mm³ isotropic voxel | FSL |
| Riemenschneider et al., 2022 | Clinical cohorts | 37±10 | 74% | AER 48 × 40 min/w, VI (n=42) | Passive (n=42) | Pre (0 w)  Mid (24 w)  Post (48 w) | 3T Siemens Skyra 3D (sequence NR) voxel size NR | FSL |
| Scheewe et al., 2013 | Clinical cohorts and Non-clinical cohorts | 28±7 | 1. 28%  2. 22% | MCE 24 × 120 min/w, MI (n=54) | 1. Active (n=23) 2. Passive (n=27) | Pre (0 w)  Post (24 w) | 3T Philips Intera 3D MPRAGE voxel size NR | SPM |
| SAVŠEK et al., 2021 | Clinical cohorts | 41±6 | 82% | AER 12 × 120 min/w, VI (n=14) | Passive (n=14) | Pre (0 w)  Post (12 w) | 3T Siemens Skyra 3D MPRAGE 1 mm³ isotropic voxel | FreeSurfer |
| Soshi et al., 2021 | Non-clinical cohorts | 73±5 | 46% | AER 12 × 90 min/w, MI (n=25) | Passive (n=25) | Pre (0 w)  Post (12 w) | 3T Siemens Verio 3D MPRAGE 1 mm³ isotropic voxel | FreeSurfer |
| Suo et al., 2016 | Clinical cohorts | 70±6 | 68% | RES 12 × 90 min/w, HI (n=19) | Active (n=21) | Pre (0 w)  Post (12 w) | 3T Siemens Trio 3D MPRAGE 1 mm³ isotropic voxel | FreeSurfer |
| Sexton et al., 2020 | Non-clinical cohorts | 66±5 | 63% | AER 12 × 90 min/w, MI (n=25) | Passive (n=25) | Pre (0 w)  Post (12 w) | 3T Siemens Prisma 3D MPRAGE 1 mm³ isotropic voxel | FreeSurfer |
| Tarumi et al., 2022 | Non-clinical cohorts | 68±5 | 75% | AER 48 × 130 min/w, VI (n=36) | Active (n=37) | Pre (0 w)  Post (48 w) | 3T Philips Ingenia 3D MPRAGE 1 mm³ isotropic voxel | FreeSurfer |
| Tarumi et al., 2019 | Clinical cohorts | 66±7 | 47% | AER 48 × 33 min/w, VI (n=16) | Active (n=20) | Pre (0 w)  Post (48 w) | 3T Philips Achieva 3D MPRAGE 1 mm³ isotropic voxel | FreeSurfer |
| Tarumi et al., 2020 | Clinical cohorts | 66±7 | 47% | AER 48 × 33 min/w, VI (n=39) | Active (n=31) | Pre (0 w)  Post (48 w) | 3T Philips Ingenia 3D MPRAGE 1 mm³ isotropic voxel | FreeSurfer |
| ten Brinke et al., 2015 | Clinical cohorts | 75±3 | 100% | 1.AER 24 × 120 min/w, VI (n=10)  2.RES 24 × 120 min/w, MI (n=8) | Active (n=11) | Pre (0 w)  Post (24 w) | 3T Philips Achieva  3D MPRAGE  1 mm³ isotropic voxel | FreeSurfer |
| Venkatraman et al., 2019 | Clinical cohorts | 73±7 | 56% | MCE 48 × 150 min/w, MI (n=50) | Passive (n=48) | Pre (0 w)  Post (48 w) | 3T Siemens Prisma 3D MPRAGE 1 mm³ isotropic voxel | FreeSurfer |
| Vidoni et al., 2021 | Non-clinical cohorts | 72±7 | 70% | AER 48 × 200 min/w, VI (n=78) | Active (n=39) | Pre (0 w)  Post (48 w) | 3T Siemens Skyra 3D MPRAGE 1 mm³ isotropic voxel | FreeSurfer |
| Vints et al., 2024 | Clinical cohorts and Non-clinical cohorts | 72±12 | 54% | RES 12 × 52 min/w, HI (n=31) | Passive (n=39) | Pre (0 w)  Post (12 w) | 3T Siemens Magnetom Prisma 3D MPRAGE 1 mm³ isotropic voxel | FreeSurfer |
| Wang et al., 2023 | Non-clinical cohorts | 66±4 | 52% | AER 48 × 180 min/w, MI (n=40) | Passive (n=42) | Pre (0 w)  Post (48 w) | 3T Siemens Prisma 3D MPRAGE 1 mm³ isotropic voxel | FreeSurfer |
| Wender et al., 2021 | Clinical cohorts | 29±2 | 40% | AER 12 × 30 min/w, MI (n=2) | Active (n=3) | Pre (0 w)  Post (12 w) | 3T Siemens (model NR) 3D (sequence NR) Voxel size NR | FreeSurfer |
| Woodward et al., 2020 | Clinical cohorts | 22±1 | 100% | 1. MBE 48 × 180 min/w, MI (n=21) 2. AER 48 × 180 min/w, MI (n=18) | Passive (n=12) | Pre (0 w)  Post (12 w) | 3T Siemens Skyra 3D MPRAGE 1 mm³ isotropic voxel | FreeSurfer |
| Wan et al., 2022 | Clinical cohorts | 66±5 | 65% | MBE 24 × 180 min/w, MI (n=51) | Passive (n=51) | Pre (0 w)  Post (24 w) | 3T Siemens Prisma 3D MPRAGE 1 mm³ isotropic voxel | FreeSurfer |
| Yu et al., 2021 | Clinical cohorts | 77±6 | 47% | AER 24 × 105 min/w, VI (n=39) | Active (n=21) | Pre (0 w)  Post (24 w)  Follow-up (48 w) | 3T Siemens Trio 3D MPRAGE 1 mm³ isotropic voxel | FreeSurfer |
| Zhu et al., 2021 | Non-clinical cohorts | 18±1 | 50% | MCE 9 × 76 min/w, VI (n=48) | Passive (n=72) | Pre (0 w)  Post (9 w) | 3T Siemens Prisma 3D MPRAGE 1 mm³ isotropic voxel | FreeSurfer |

Note: NR = Not reported; AER = Aerobic exercise; RES = Resistance exercise: MBE = Mind-body exercise: MCE = Multicomponent exercise; HI = High intensity; LI = Low intensity; MI = Moderate intensity; VI= Vigorous intensity; UI = Unspecific intensity.

## Table S7. Risk of bias summary.

| Study | Random sequence generation (selection bias) | Allocation concealment (selection bias) | Blinding of participants and personnel (performance bias) | Blinding of outcome assessment (detection bias) | Incomplete outcome data (attrition bias) | Selective reporting (reporting bias) | Other sources of bias |
| --- | --- | --- | --- | --- | --- | --- | --- |
| Best et al., 2015 | Unclear | Unclear | High | Low | Low | Low | Low |
| Boecker et al., 2024 | Unclear | Unclear | Unclear | Unclear | Unclear | Unclear | Unclear |
| Broadhouse et al., 2020 | Low | Low | Low | Low | Low | High | Low |
| Blackmore et al., 2024 | Low | Low | High | High | Low | Low | Low |
| Bergman et al., 2020 | Unclear | Unclear | Unclear | Unclear | Low | Low | Low |
| Callisaya et al., 2017 | Low | Low | Unclear | Low | Low | Low | Low |
| Castells-Sánchez et al., 2022 | Low | Low | Unclear | Low | Low | Low | Low |
| Demnitz et al., 2022 | Unclear | Unclear | High | Low | Low | Low | Low |
| Damme et al., 2022 | Unclear | Unclear | Unclear | Low | Low | Low | Low |
| Erickson et al., 2011 | Unclear | Unclear | Unclear | Unclear | Low | Low | Low |
| Ehlers et al., 2017 | Low | Unclear | High | Low | Low | Low | Low |
| Frost et al., 2022 | Low | Unclear | High | Low | Low | Low | Low |
| Frederiksen et al., 2018 | Unclear | Unclear | High | Low | Low | Low | Low |
| Frodl et al., 2020 | Low | Unclear | Unclear | Unclear | Low | Low | Low |
| Feys et al., 2019 | Unclear | Unclear | Unclear | Unclear | Low | Low | Low |
| Gylling et al., 2024 | Low | Low | High | Low | Low | Low | Low |
| Guzman et al., 2021 | Unclear | Unclear | Unclear | Unclear | Low | Unclear | Unclear |
| Jonasson et al., 2017 | Unclear | Unclear | Unclear | Unclear | Unclear | Low | Low |
| Kaiser et al., 2022 | Unclear | Unclear | High | Unclear | Unclear | Low | Unclear |
| Krogh et al., 2014 | Low | Low | High | Low | High | Low | Low |
| Koevoets et al., 2023 | Low | Low | Unclear | Unclear | Low | Low | Low |
| Lin et al., 2015 | Low | Low | High | Low | Low | Low | Low |
| Lenze et al., 2022 | Low | Unclear | High | Low | Low | Low | Low |
| Liu et al., 2019 | Unclear | Unclear | Unclear | Unclear | Low | Unclear | Low |
| Liu-Ambrose et al., 2010 | Low | Low | High | Low | Low | Low | Low |
| Liu et al., 2021 | Unclear | Unclear | Unclear | Unclear | Low | Unclear | Unclear |
| Langeskov-Christensen et al., 2021 | Low | Low | High | Low | Low | Low | Low |
| Mortimer et al., 2012 | Low | Low | High | Low | Low | Low | Unclear |
| Morris et al., 2017 | Low | Unclear | High | Low | Low | Low | Low |
| Maurer et al., 2022 | Low | Low | High | High | Low | Low | Low |
| Malchow et al., 2015 | Unclear | Unclear | Unclear | Low | Low | Low | Low |
| Matura et al., 2017 | Low | Unclear | High | Low | Low | Low | Low |
| Niemann et al., 2014a | Unclear | Unclear | Unclear | Unclear | Unclear | Unclear | Low |
| Niemann et al., 2014b | Unclear | Unclear | Unclear | Low | Unclear | Low | Low |
| Nagamatsu et al., 2016 | Low | Unclear | High | Low | Low | Low | Low |
| Ortega et al., 2022 | Low | Low | High | High | Low | Low | Low |
| Pajonk et al., 2010 | Low | Unclear | Unclear | Low | Low | Low | Low |
| Pani et al., 2021 | Low | Unclear | Unclear | Unclear | Low | Low | Low |
| Rocca et al., 2024 | Low | Low | High | Low | Low | Low | Unclear |
| Rosano et al., 2017 | Low | Unclear | Unclear | Low | Low | Low | Low |
| Riemenschneider et al., 2022 | Low | High | Low | Low | High | Low | Low |
| Scheewe et al., 2013 | Low | Low | Unclear | Low | Low | Low | Low |
| SAVŠEK et al., 2021 | Unclear | Unclear | Unclear | Unclear | Low | Low | Low |
| Soshi et al., 2021 | Unclear | Unclear | Unclear | Unclear | Low | Low | Low |
| Suo et al., 2016 | Low | Unclear | High | Low | Low | Low | Low |
| Sexton et al., 2020 | Low | Low | High | Low | Low | Low | Low |
| Tarumi et al., 2022 | Low | Low | High | Low | Low | Low | Low |
| Tarumi et al., 2019 | Low | Low | High | Low | Low | Low | Low |
| Tarumi et al., 2020 | Low | Unclear | High | Low | High | Unclear | Unclear |
| ten Brinke et al., 2015 | Unclear | Unclear | High | Low | Low | Low | Low |
| Venkatraman et al., 2019 | Low | Unclear | High | Low | Low | Low | Low |
| Vidoni et al., 2021 | Low | Unclear | Unclear | Low | Low | Low | Low |
| Vints et al., 2024 | Low | Low | High | Low | Low | Low | Low |
| Wang et al., 2023 | Low | Low | High | Low | Low | Low | Low |
| Wender et al., 2021 | Unclear | Unclear | Unclear | Unclear | Unclear | Unclear | Unclear |
| Woodward et al., 2020 | Low | Low | High | Low | High | Low | Low |
| Wan et al., 2022 | Low | Low | High | Low | Low | Low | Low |
| Yu et al., 2021 | Low | Low | High | Low | Low | Low | Low |
| Zhu et al., 2021 | Unclear | Unclear | Unclear | Unclear | Low | Low | Low |

|  |
| --- |


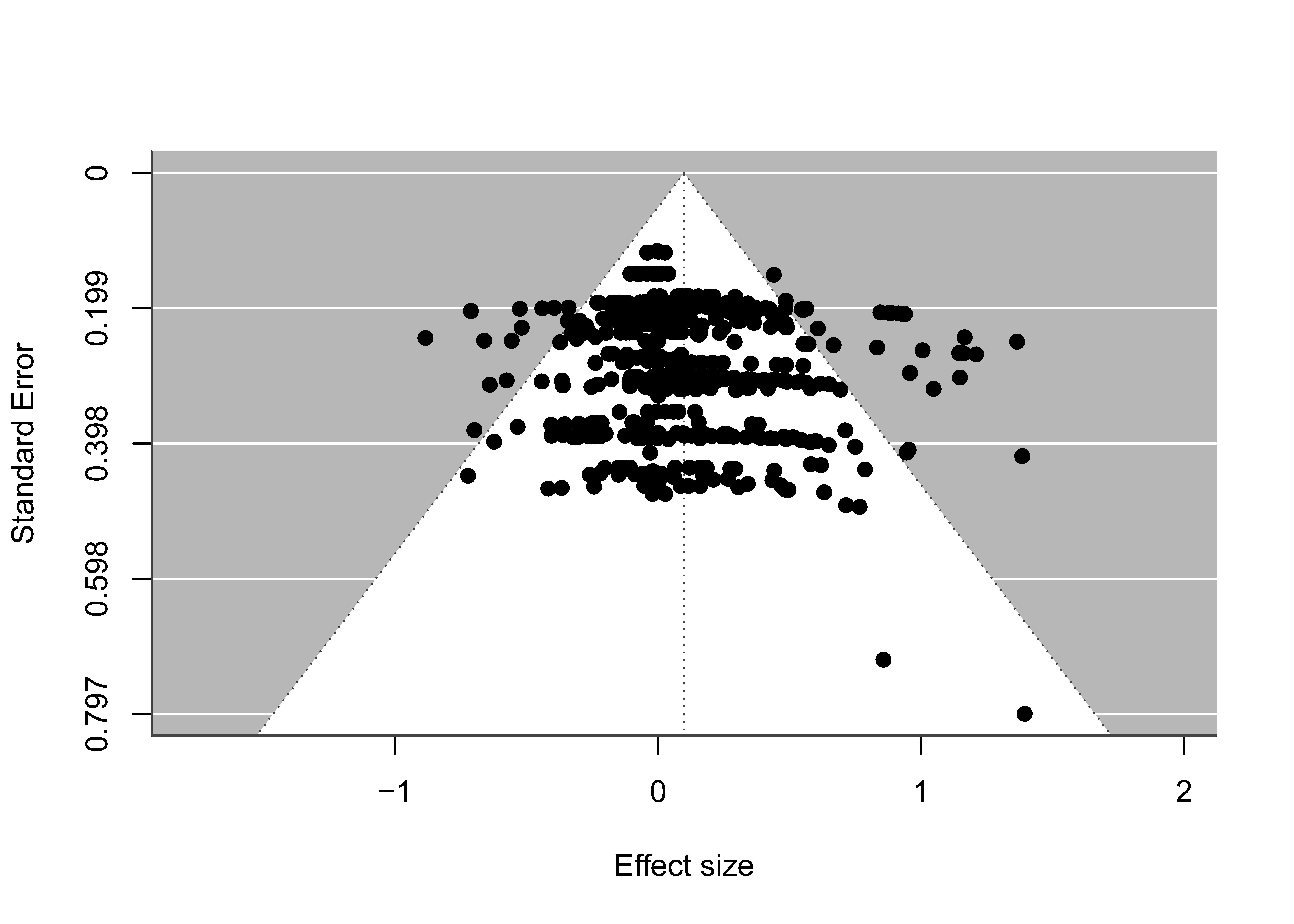


**Figure S1.** Funnel plot.


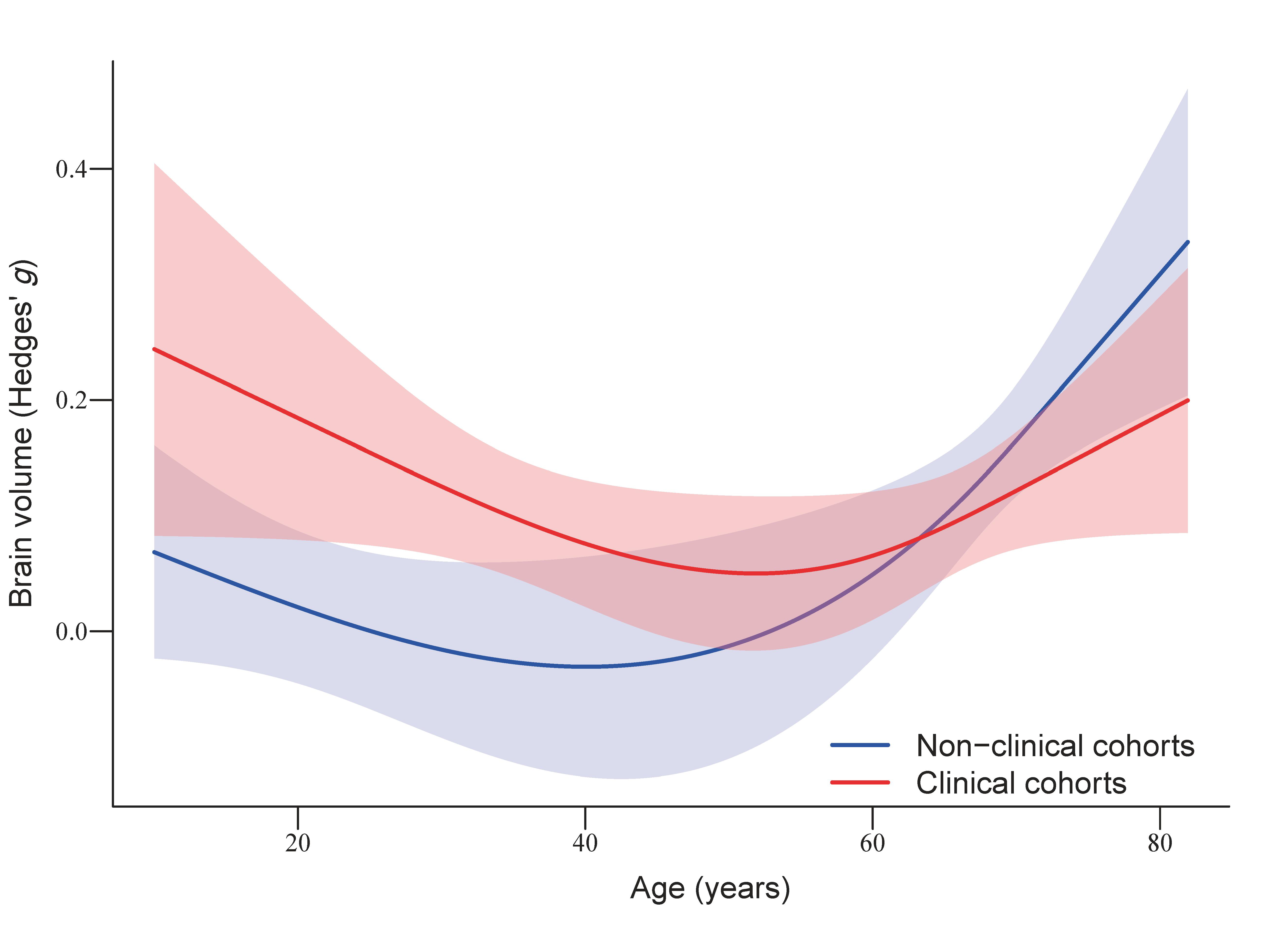


**Figure S2.** Sensitivity analysis of dose-response relationships stratified by clinical versus non-clinical cohorts.

**Reference**

1. Fjell AM, Walhovd KB. Structural brain changes in aging: courses, causes and cognitive consequences. Rev Neurosci. 2010;21(3):187–221. doi:10.1515/revneuro.2010.21.3.187
2. Erickson KI, Raji CA, Lopez OL, Becker JT, Rosano C, Newman AB, et al. Physical activity predicts gray matter volume in late adulthood: the Cardiovascular Health Study. Neurology. 2010;75(16):1415–22. doi:10.1212/WNL.0b013e3181f88359
3. Stillman CM, Esteban-Cornejo I, Brown B, Bender CM, Erickson KI. Effects of exercise on brain and cognition across age groups and health states. Trends Neurosci. 2020;43(7):533–543. doi:10.1016/j.tins.2020.04.010
4. Gogniat MA, Robinson TL, Miller LS. Exercise interventions do not impact brain volume change in older adults: A systematic review and meta-analysis. Neurobiol Aging. 2021;101:230–246.
5. Van den Noortgate W, López-López JA, Marín-Martínez F, Sánchez-Meca J. Three-level meta-analysis of dependent effect sizes. Behav Res Methods. 2013;45:576–594.
6. Assink M, Wibbelink CJ. Fitting three-level meta-analytic models in R: A step-by-step tutorial. Quant Methods Psychol. 2016;12:154–174.
7. Higgins C, Smith BH, Matthews K. Evidence of opioid-induced hyperalgesia in clinical populations after chronic opioid exposure: A systematic review and meta-analysis. Br J Anaesth. 2019;122(6). doi:10.1016/j.bja.2019.03.033
8. Viechtbauer W. Conducting meta-analyses in R with the metafor package. J Stat Softw. 2010;36(3):1–48.
9. Ludyga S, Gerber M, Pühse U, et al. Systematic review and meta-analysis investigating moderators of long-term effects of exercise on cognition in healthy individuals. Nat Hum Behav. 2020;4:603–612.
10. Balbim GM, Boa Sorte Silva NC, Ten Brinke L, Falck RS, Hortobágyi T, Granacher U, et al. Aerobic exercise training effects on hippocampal volume in healthy older individuals: a meta-analysis of randomized controlled trials. GeroScience. 2024;46(2):2755–2764. doi:10.1007/s11357-023-00971-7
11. Tully J, Cross B, Gerrie B, et al. A systematic review and meta-analysis of brain volume abnormalities in disruptive behaviour disorders, antisocial personality disorder and psychopathy. Nat Ment Health. 2023;1(3):163–173.
12. Xiaolan M, Duan Z, Niu Z, Jiang J, Wei X, Chen X. Non-linear associations between night shifts and adverse events in nursing staff: A restricted cubic spline analysis. BMC Nurs. 2024;23(1):602. doi:10.1186/s12912-024-02259-3
13. Firth J, Stubbs B, Vancampfort D, Firth J. Effect of aerobic exercise on hippocampal volume in humans: A systematic review and meta-analysis. NeuroImage. 2018;166:230–238.
14. Egger M, Smith GD, Schneider M, Minder C. Bias in meta-analysis detected by a simple, graphical test. BMJ. 1997;315(7109):629–634.
15. Caspersen CJ, Powell KE, Christenson GM. Physical activity, exercise, and physical fitness: definitions and distinctions for health-related research. Public Health Rep. 1985;100(2):126–131.
16. Norton K, Norton L, Sadgrove D. Position statement on physical activity and exercise intensity terminology. J Sci Med Sport. 2010;13(5):496–502.
